# Dynamic Evolution of Quantitative Electroencephalography During Graded Intracranial Hypertension and Screening of Candidate Early-Warning Features in a Rabbit Model

**DOI:** 10.3390/brainsci16101116

**Published:** 2026-10-16

**Authors:** Jianxiang Zhao, Yang Zhang, Kaiyu Wang, Feng Chen, Han Chen

**Affiliations:** 1 Shengli Clinical Medical College of Fujian Medical University, Fuzhou 350001, China; 2 Department of Critical Care Medicine, Fujian Provincial Hospital, Fuzhou 350001, China; 3 Fuzhou University Affiliated Provincial Hospital, Fuzhou 350001, China; 4 Fujian Provincial Key Laboratory of Emergency Medicine, Fuzhou 350001, China; 5 Department of Neurosurgery, Fujian Provincial Hospital, Fuzhou 350001, China; 6 Department of Emergency, Fujian Provincial Hospital, Fuzhou 350001, China

**Keywords:** intracranial pressure, quantitative electroencephalography, amplitude-integrated electroencephalography, EEG spectral band power, candidate early-warning feature, rabbit

## Abstract

**Highlights:**

**What are the main findings?**

- The aEEG lower margin and θ power changed earliest and most consistently as ICP rose within this cohort.
- qEEG changes accompanied a falling cerebral perfusion pressure, although ICP and CPP effects could not be separated.

**What are the implications of the main findings?**

- The aEEG lower margin and θ power are candidate bedside early-warning features for intracranial hypertension.
- These exploratory, small-cohort findings require confirmation in larger studies.

**Abstract:**

**Background and Objectives:** Elevated intracranial pressure (ICP) is common to many encephalopathies, but the evolution of quantitative electroencephalography (qEEG) indices along a pressure gradient—and their relative suitability for early warning—have not been systematically compared. In this exploratory study we established a stepwise, steady-state rabbit model of graded intracranial hypertension and screened candidate early-warning features against prespecified criteria. **Materials and Methods:** Eight male New Zealand rabbits were studied; two were excluded—one for circulatory failure during catheterization and one for refractory status epilepticus that reached a humane endpoint—leaving six that completed the protocol. Repeated small-volume inflation of a left parenchymal water balloon was used to define five windows: baseline (BL; ICP < 10 mmHg), 15–20, 25–30 and 35–40 mmHg, and an end stage (END). Eight-channel EEG was recorded synchronously, and δ, θ, α and β power, together with the amplitude-integrated EEG (aEEG) upper margin, lower margin and bandwidth, were computed. Seven candidate features and three criteria (evolution timing, individual consistency and strength of association with ICP) were fixed before analysis; selection stability was assessed by leave-one-rabbit-out (LORO) cross-validation. **Results:** ICP rose in a controlled manner from 5.43 ± 1.09 to 35.93 ± 1.48 mmHg (Kendall W = 0.96), without systematic changes in respiratory mechanics. As ICP increased, absolute θ and α power declined progressively (ρ = −0.77 and −0.53), whereas absolute δ power did not increase and its rising relative share merely reflected redistribution after the loss of faster activity. The three aEEG components changed asynchronously: the lower margin shifted downward early and persistently (ρ = −0.87 with ICP; W = 0.86), whereas the upper margin changed only terminally and bandwidth showed no consistent direction. Concurrently, MAP rose through the Cushing response, with heart-rate slowing, but the rise was insufficient and CPP fell from 64.78 to 41.24 mmHg, approaching the rabbit lower limit of autoregulation (~50 mmHg CPP) only in the later windows. Because ICP and CPP were tightly coupled within animals, their independent effects on the EEG could not be separated. In leave-one-rabbit-out cross-validation, the lower margin was selected in five of six folds, with a median out-of-sample ρ of −1.00 (a discrete value based on four windows per animal). **Conclusions:** In this exploratory study in a small cohort (*n* = 6), qEEG indices evolved asynchronously along the ICP gradient; the aEEG lower margin appeared earliest and remained relatively stable across leave-one-rabbit-out folds, making it a candidate early-warning feature worthy of priority validation. However, because rising ICP and falling CPP were tightly coupled and could not be separated in this model, its early decline cannot be uniquely attributed to either ICP elevation or hypoperfusion. Its alarm threshold and early-warning value still require confirmation in larger independent cohorts with simultaneous CPP and cerebral blood flow monitoring.

## 1. Introduction

Elevated intracranial pressure (ICP) is a common pathological stage in many encephalopathies, including traumatic brain injury and stroke. Under the Monro–Kellie framework, once compensatory mechanisms are exhausted and ICP rises abruptly, cerebral perfusion pressure (CPP) falls and secondary brain injury is aggravated; continuous monitoring and early recognition are therefore central to the management of intracranial hypertension [1,2]. Quantitative electroencephalography (qEEG), derived from continuous EEG (cEEG), is non-invasive, lends itself to continuous bedside monitoring and has been increasingly used in neurocritical care [3,4,5]. Because EEG is highly sensitive to cerebral blood flow, attenuation of fast activity and EEG slowing often precede a rise in ICP, cerebral herniation, and changes in consciousness or pupils. Quantitative trends can detect cortical hypoperfusion before irreversible injury and qEEG has been proposed as a potential early-warning tool [3,4,5]. Nevertheless, the parameters best suited to early warning—and their relative performance—remain unclear.

Two families of qEEG trend variables are in common use: the absolute and relative power of the δ, θ, α and β bands (spectral parameters), and the upper margin, lower margin and bandwidth of amplitude-integrated EEG (aEEG; amplitude parameters). How these indices evolve along an ICP gradient and which is best suited as an early-warning feature have not been systematically compared within a single model for the following three reasons. First, clinical observations are largely anecdotal or cross-sectional: patients are heterogeneous and pressure uncontrolled, making it difficult to disentangle pressure level from inflation speed and inter-individual variability [6]. Second, existing animal models generally rely on a single rapid inflation and contrast only “normal” with “circulatory arrest”, lacking the intermediate pressure gradients required to trace evolution [7]. Third, earlier studies usually related a single spectral index to ICP [8] without resolving the temporal order and individual consistency of the components; relative power is also susceptible to a “denominator effect” caused by the loss of fast activity, while evidence on aEEG under graded intracranial hypertension remains scarce. We therefore asked the following research question: over a pressure-controlled course of graded intracranial hypertension, in what temporal order do the various qEEG indices evolve, and which can be prioritized as candidate early-warning features?

To address this question, we established a stepwise, steady-state rabbit model of graded intracranial hypertension, using as each animal’s baseline its stabilized state immediately before the first inflation. Along the ICP gradient, we systematically characterized the dynamic evolution of δ, θ, α and β power and of the aEEG margins and bandwidths, compared their timing, inter-individual consistency and strength of association with ICP, and—applying prespecified criteria—screened candidate early-warning features, whose stability was then tested by leave-one-rabbit-out (LORO) cross-validation. Our aim was to provide candidate features and experimental evidence to support the use of qEEG in continuous bedside monitoring of intracranial hypertension; given the small sample, these features remain exploratory and await confirmation in an independent cohort.

## 2. Materials and Methods

### 2.1. Animals, Ethics and Experimental Design

This was a single-group, longitudinal, repeated-measures animal study. Eight conventional-grade male New Zealand rabbits, aged 3.5 months and weighing 2.42–2.55 kg, were obtained from Songlian Experimental Animal Farm (Songjiang District, Shanghai, China; Animal Production License No. SCXK (Hu) 2022-0006) and housed at 22–25 °C under a 12 h light/dark cycle with free access to food and water. The protocol was approved by the Institutional Animal Care and Use Committee (IACUC) of Fujian Medical University (IACUC FJMU 2022-0641; date of approval: 9 June 2022), and the study is reported in accordance with the ARRIVE 2.0 guidelines [9]; the completed ARRIVE 2.0 Author Checklist is provided as Supplementary Material S1. Because this was an exploratory study with no prior variance data for the present model, a formal a priori power calculation was not feasible; *n* = 6 was prespecified based on comparable large-animal intracranial hypertension studies [10] and on feasibility, and is adequate for the exploratory, hypothesis-generating purpose of this work. Of the eight animals, one developed circulatory failure during catheterization and one reached a humane endpoint through refractory status epilepticus, and both were excluded, leaving six animals that completed the study. The order of stages was not randomized because ICP had to rise in a stepwise manner; balloon inflation and EEG acquisition were separated from data analysis, and the statistical analysis was performed in a blinded manner by an investigator who was not involved in the procedures. The state immediately before the first inflation served as the self-baseline (BL; ICP < 10 mmHg); no separate control group was used. The five ICP windows constituted within-animal repeated measures, whereas the eight channels were descriptive and were not treated as independent replicates. Seven candidate features were prespecified—δ, θ, α and β band power and the aEEG upper margin, lower margin and bandwidth—to be screened against three criteria: evolution timing, individual consistency (quantified by Kendall W) and strength of association with ICP (quantified by Spearman ρ). The study was designed as a screening of candidate features.

At the end of the experiment or upon reaching a humane endpoint (circulatory failure or refractory status epilepticus), approximately twice the anesthetic dose of ethyl carbamate was first administered intravenously; after the corneal reflex had disappeared (deep anesthesia), 10 mL of 10% potassium chloride was rapidly injected intravenously to induce cardiac arrest.

### 2.2. Anesthesia, Ventilation and Multi-Parameter Monitoring

**Figure 1:**
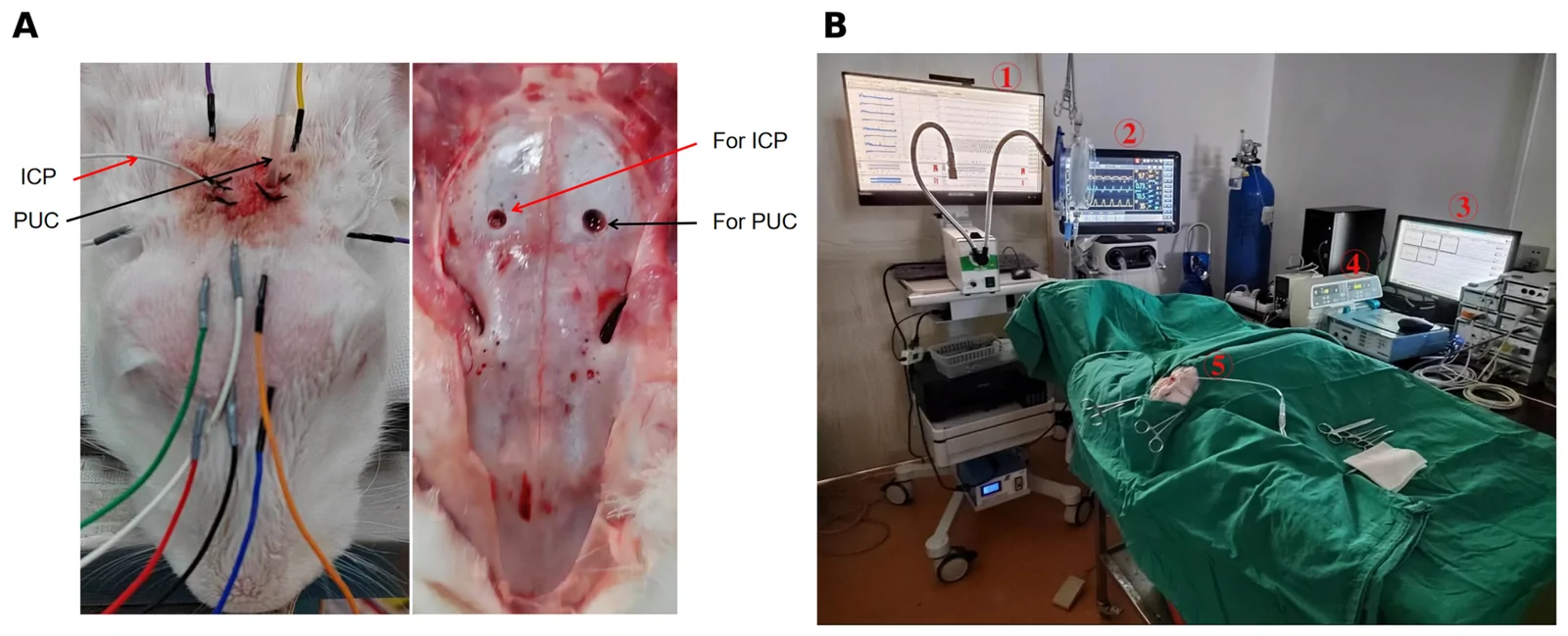
Placement of EEG electrodes, the ICP probe and the water-balloon catheter, and the multi-parameter monitoring system. (**A**) In vivo placement of EEG electrodes, the ICP probe and the water-balloon catheter (pediatric urinary catheter, PUC) and the cranial burr holes; the red and black arrows indicate the ICP probe and the water-balloon catheter, respectively; the colored leads are the eight EEG channels (F3/F4, C3/C4, P3/P4, T3/T4), and the black and white leads are the ground and reference electrodes. (**B**) The multi-parameter monitoring setup: ① quantitative EEG monitor, ② ventilator, ③ biosignal acquisition system, ④ syringe pump, ⑤ operating table and surgical instruments.

The monitoring equipment comprised an EB Neuro qEEG system, an SV800 ventilator, a Codman ICP monitor and PowerLab/LabChart (Figure 1B). After induction with isoflurane, 20% ethyl carbamate (1 g/kg) was administered intraperitoneally, and anesthesia was maintained through a femoral venous line at 3 mg/(kg·min), titrated to a spectral entropy of 40–60; a femoral artery line was used to measure mean arterial pressure (MAP) and heart rate (HR); body temperature was maintained at 38.5–39.0 °C with a heating pad and monitored continuously throughout the experiment. A tracheostomy was connected to the ventilator at 40 breaths/min, a tidal volume of 4–7 mL/kg, a fraction of inspired oxygen (FiO_2_) of 21%, a positive end-expiratory pressure (PEEP) of 3 cmH_2_O and an inspiratory-to-expiratory ratio of 1:2, with fine adjustment guided by end-tidal carbon dioxide (EtCO_2_). ICP, circulatory, respiratory and EEG variables were acquired synchronously (Figure 1). The ICP probe was inserted 1 cm into the parenchyma through a 1.5 mm burr hole placed 5 mm to the right of the midline and 5 mm behind the coronal suture, and was sampled at 400 Hz. Ten needle electrodes formed eight bipolar channels (Figure 1A), with impedance <5 kΩ, a band-pass of 0.3–60 Hz and a 50 Hz notch filter.

### 2.3. Construction of the Graded Intracranial Hypertension Model

**Figure 2:**
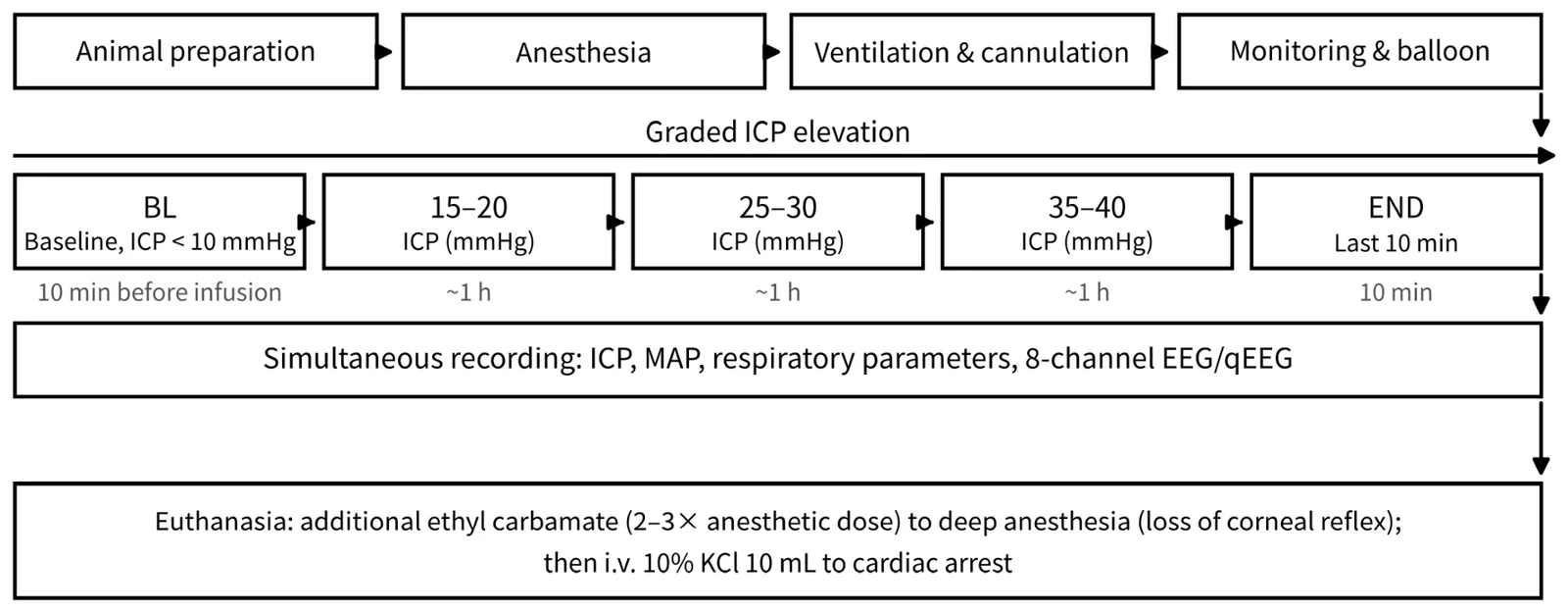
Experimental flowchart.

**Figure 4:**
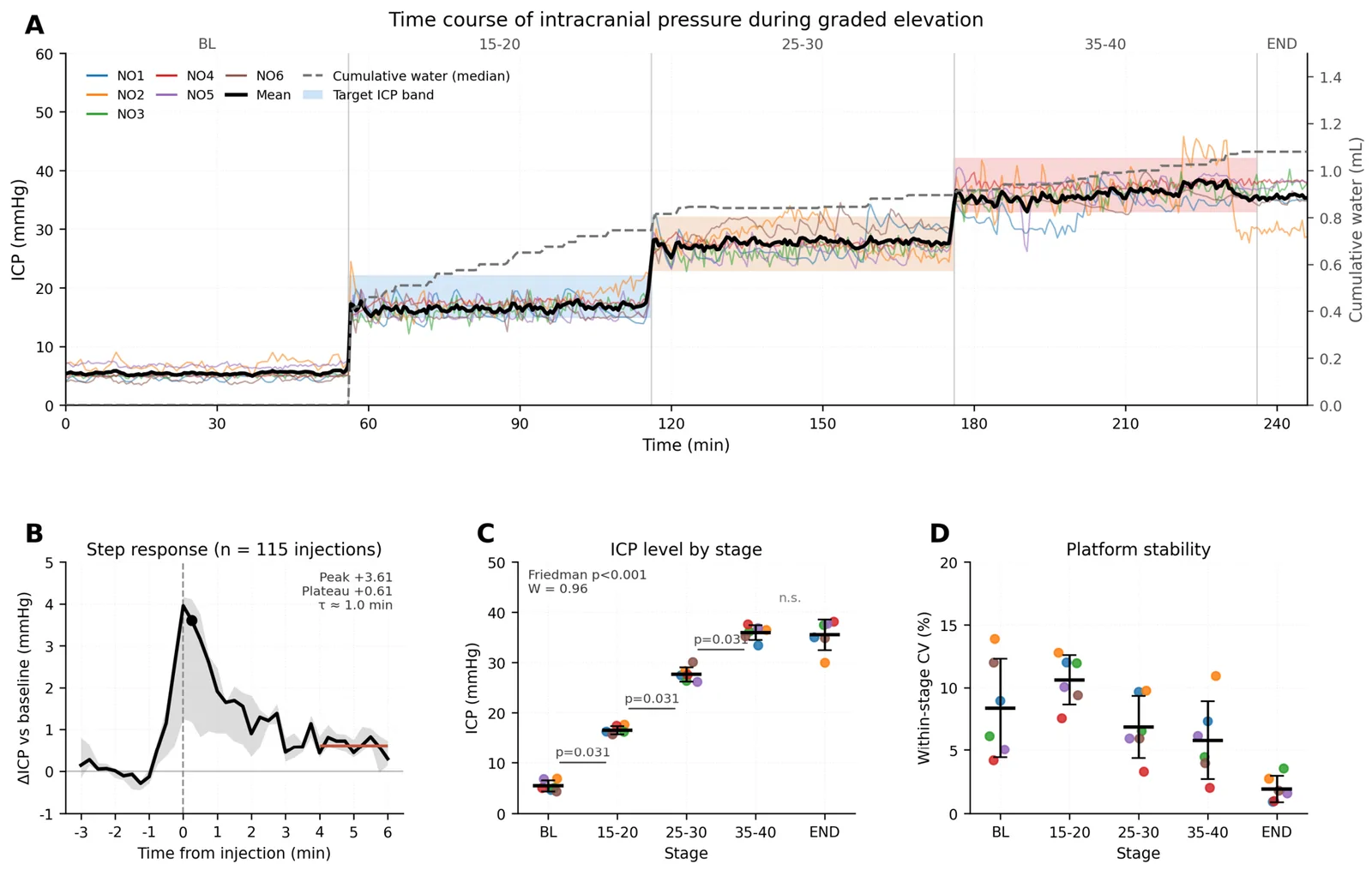
Construction of the stepwise steady-state model and graded elevation of ICP (*n* = 6). (**A**) Stage-aligned ICP time curves: thin lines—individual rabbits; thick black line—mean; gray dashed line (right axis)—median cumulative inflation volume; shaded bands—target gradient ranges. (**B**) Step response after inflation (115 events aligned at t = 0): black line—median ΔICP; gray band—interquartile range. (**C**) ICP level at each stage (horizontal line—mean; error bars—SD), with paired Wilcoxon *p* values; n.s.—not significant. (**D**) Plateau stability (within-stage CV) at each stage.

Following established models [11,12], a burr hole was drilled 5 mm paramedian and posterior to the left coronal suture, and a water-balloon catheter (a 6 Fr pediatric urinary catheter, PUC; outer diameter 1.98 mm) was placed in the left temporoparietal region. Once BL (ICP < 10 mmHg) had been reached, 0.9% saline was injected into the balloon in aliquots of 0.1–0.5 mL; after each aliquot, the brief pressure jump settled within about 1 min (Figure 4B). Because ICP could drift downward over time, repeated small aliquots were added as needed to keep ICP within the target range so that ICP was maintained successively at 15–20, 25–30 and 35–40 mmHg for approximately 1 h each. The end stage (END) was defined as the final 10 min; together with BL, this yielded five time windows (Figure 2).

### 2.4. EEG Signal Processing and Quantitative Analysis

Two complementary families of qEEG indices were analyzed—spectral parameters describing frequency composition and amplitude parameters reflecting the overall fluctuation of the background activity—both of which remain continuously readable at the bedside. (i) Spectral parameters: after fast Fourier transformation using a 20 s Hanning window with 50% overlap, absolute power and its relative share of total power were computed for δ (0.5–4 Hz), θ (4–8 Hz), α (8–13 Hz) and β (13–30 Hz); segments showing electrode saturation, impedance drift or marked electromyographic/movement artifact were excluded, and within each window the remaining stable 20 s segments were averaged across all eight channels. Because channel power spanned several orders of magnitude, the animal-level within-window power was obtained as the geometric mean across channels, whereas the aEEG upper and lower margins and bandwidth were obtained as arithmetic means. (ii) Amplitude parameters: following the original cerebral function monitoring method and its modern refinements, after 0.5–35 Hz band-pass filtering the EEG was rectified, smoothed and time-compressed with a semi-logarithmic amplitude display (linear below 10 μV and logarithmic above), producing an aEEG trend band with a 2 s time constant and a 0.5 μV lower bound, from which the upper and lower margins were extracted; bandwidth was defined as their difference.

### 2.5. Statistical Analysis

Non-parametric statistics were used, with inference at the animal level (*n* = 6); the eight channels were descriptive only and were not treated as independent replicates. Because *n* = 6 is too small to assess normality reliably, non-parametric tests were used; data are expressed as mean ± standard deviation (SD). Dispersion and stage effects were quantified by the coefficient of variation (CV) and eta-squared (η^2^). Differences across the five windows were tested using Friedman’s test, with Kendall’s coefficient of concordance W (a 0–1 measure of agreement, where 1 indicates identical ranking across animals) reported alongside; pairwise comparisons used Wilcoxon signed-rank tests with Holm correction, and trends used Page’s test. Because the relative band powers are compositional (they sum to one), and therefore cannot be analyzed independently, they underwent centered log-ratio (CLR) transformation before testing. Effect sizes are reported as geometric-mean ratios relative to BL, with 95% confidence intervals (CIs) obtained by rabbit-clustered bootstrap. Each index was correlated with ICP (window medians; END excluded) using Spearman’s rank correlation; per-animal ρ was computed over the four windows and then averaged across animals by direct arithmetic averaging (a Fisher-z-transformed average was also evaluated as a sensitivity analysis). CPP was derived at each time point as MAP−ICP, and each feature was likewise correlated with CPP and MAP and averaged in the same way. To test whether ICP and CPP carried independent information for the lower margin, within-animal partial correlations (controlling for each other) were computed together with variance inflation factors. Ventilator and hemodynamic variables, which are stable over minute-long timescales, were recorded at 1 min intervals and time-aligned with ICP (*N* = 1426); the continuous EEG from which the qEEG indices were derived was sampled at 400 Hz throughout. Independent effects of respiratory and circulatory variables on ICP were estimated with fixed-effects (rabbit-indicator) models on these time-aligned data, controlling for MAP and HR, with rabbit-clustered robust standard errors, and quantified by semipartial R^2^. The eight-channel *p* values were corrected using the Benjamini–Hochberg false-discovery-rate (FDR) procedure. All seven candidate features and their selection criteria were fixed before analysis and were neither added nor removed. To test whether feature selection depended on any particular animal, leave-one-rabbit-out (LORO) cross-validation was used: in each of six folds, one rabbit was held out as the test animal, the optimal feature was selected on the remaining five using the three prespecified criteria and its Spearman correlation with ICP (the out-of-sample ρ) was evaluated in the held-out rabbit. All tests were two-sided, with α = 0.05. Analyses were performed in Python 3.8.

## 3. Results

### 3.1. Multi-Parameter Monitoring and Stability of Physiological Variables

At baseline, before ICP was raised, ICP, MAP, CPP, HR, the respiratory-mechanics variables (Pmean, PEEP, EtCO_2_, tidal volume and respiratory rate) and body temperature were monitored synchronously (Table 1). Baseline values were closely matched across animals: baseline ICP was 5.43 ± 1.09 mmHg and, because the mean was low, its SD of about 1 mmHg translated into a CV of 20.07%, whereas the between-animal CV of every other variable was below 11%.

**Figure 3:**
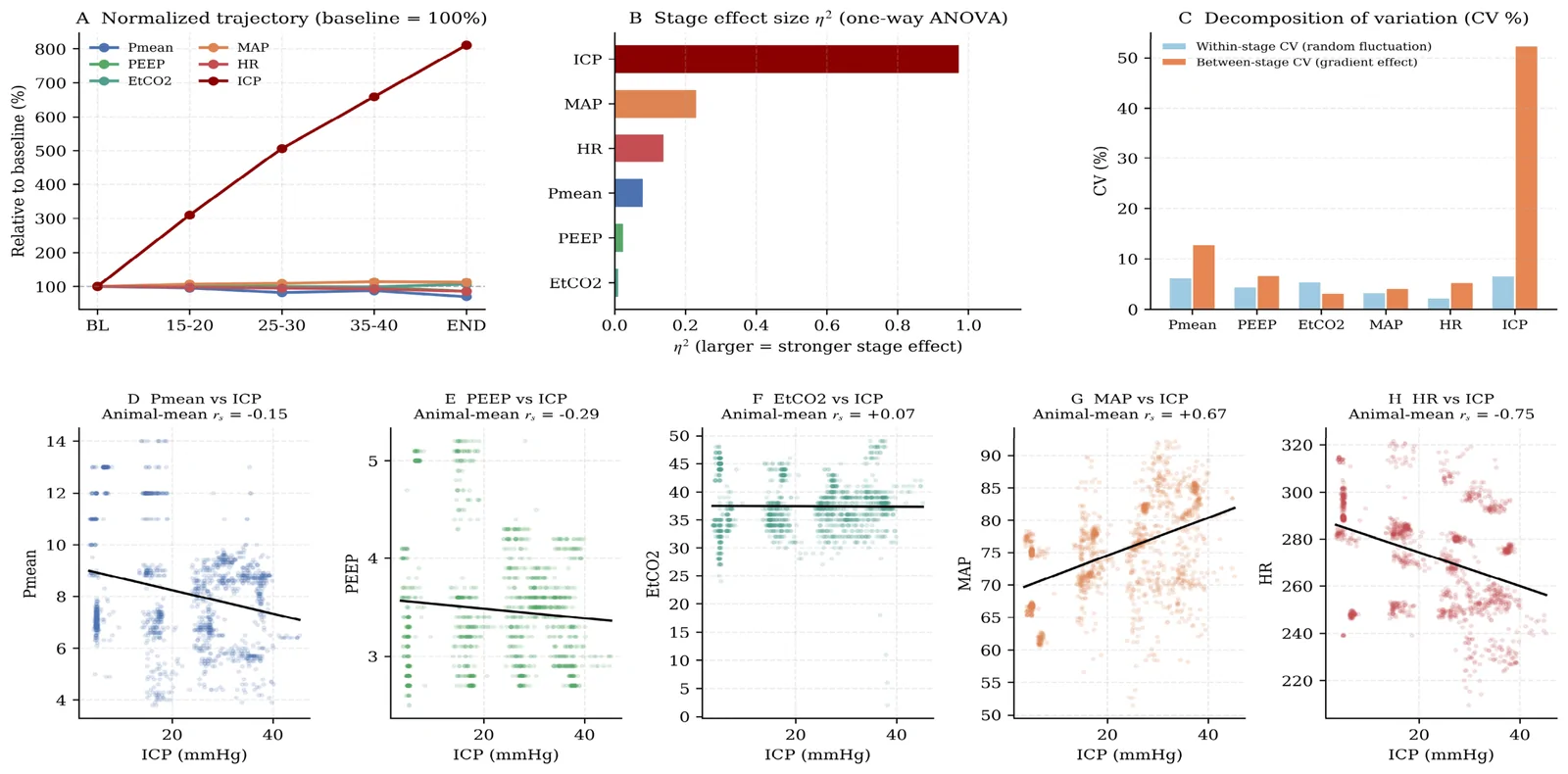
Stability of respiratory and circulatory parameters and their independence from ICP evolution during graded intracranial hypertension (*n* = 6). (**A**) Trajectories normalized to baseline (BL = 100%). (**B**) Stage effect sizes η^2^ (one-way analysis of variance, ANOVA). (**C**) Decomposition of within-/between-stage CV. (**D**–**H**) Scatter plots against ICP with least-squares fits, in the order (**D**) Pmean, (**E**) PEEP, (**F**) EtCO_2_, (**G**) MAP, (**H**) HR; the animal-level Spearman correlation is shown in the upper-right corner.

To rule out the possibility that mechanical ventilation and circulatory fluctuations confounded ICP and its EEG interpretation, Figure 3 examines whether respiratory and circulatory variables co-varied with the ICP gradient. None of the three respiratory-mechanics variables changed systematically with ICP: their stage effects were small (η^2^ = 0.079, 0.023 and 0.010; Figure 3B), they were virtually uncorrelated with ICP (ρ = −0.15, −0.29 and +0.07; Figure 3D–F), and both components of their variation remained small, with within-stage CVs of 6.2%, 4.4% and 5.3% and between-stage CVs of 12.8%, 6.7% and 3.1%, in marked contrast to the between-stage CV of ICP (52.2%; Figure 3C). MAP and HR were more strongly associated with ICP (ρ = +0.67 and −0.75; Figure 3G,H), but in the direction expected from “rising ICP → falling CPP → circulatory compensation”, i.e., as a consequence rather than a cause. In a fixed-effects model with rabbit indicators that controlled for MAP and HR (*N* = 1426, rabbit-clustered robust standard errors), none of the three respiratory variables exerted a significant independent effect on ICP (Pmean coefficient −2.37, 95% CI −5.40 to 0.66, *p* = 0.100; PEEP −1.45, 95% CI −10.60 to 7.71, *p* = 0.702; EtCO_2_ −0.46, 95% CI −1.49 to 0.58, *p* = 0.307), together accounting for 7.4% of ICP variance (semipartial R^2^), whereas MAP (*p* = 0.020) and HR (*p* = 0.010) were significant. A simple within-animal check led to the same conclusion: from baseline to the highest stage, the three respiratory variables did not change in a consistent direction (Pmean and PEEP increased in 2/6 and decreased in 4/6 rabbits; EtCO_2_ increased in 3/6 and decreased in 3/6), as also seen in the individual trajectories of Figure 3A. These results provide no evidence that respiratory or circulatory fluctuations drove the graded ICP elevation, which was governed primarily by the inflation volume; however, because ICP and experimental time co-varied and no sham-operated group was included, the effects of anesthesia and cumulative time cannot be fully excluded (see Section 4.5).

### 3.2. Construction of the Graded Model and Dynamic Changes in ICP

Repeated inflation of the intraparenchymal balloon reliably established a controllable, stepwise steady-state graded intracranial hypertension (Figure 4A). ICP rose from a baseline of 5.43 ± 1.09 mmHg to 16.49 ± 0.82, 27.63 ± 1.44 and 35.93 ± 1.48 mmHg; adjacent levels differed significantly (all *p* = 0.031; overall *p* < 0.001; W = 0.96). END was maintained at 35.49 ± 3.06 mmHg and did not differ from the 35–40 mmHg level (*p* = 0.563), confirming stable maintenance once the target was reached. Each of the three levels was maintained for approximately 1 h (57.1 ± 13.8, 64.1 ± 14.6 and 61.9 ± 26.2 min), and END comprised the final 10 min.

The stepwise rise was achieved through the accumulation of small-volume injections and was smooth (Figure 4B): all 115 injections produced only brief, small jumps (median peak increment 3.61 mmHg, with ICP returning to a stable level within about 1.0 min and the new plateau lying only 0.61 mmHg higher); a cumulative volume of 0.51–1.40 mL (median 1.08 mL) attained the target without loss of pressure control. Plateau stability improved progressively across the pressure levels (Figure 4C,D): from the 15–20 mmHg plateau to END, the within-stage CV decreased stepwise (10.61% ± 1.98%, 6.85% ± 2.48%, 5.80% ± 3.11% and 1.92% ± 1.03% at the 15–20, 25–30 and 35–40 mmHg plateaus and at END, respectively). The time required to reach 35 mmHg ranged from 136 to 214 min across rabbits, reflecting inter-individual differences in intracranial compensatory reserve.

### 3.3. Dynamic Evolution and Power Analysis of the EEG Under Graded Intracranial Hypertension

#### 3.3.1. Gradual Evolution of EEG Waveforms

**Figure 5:**
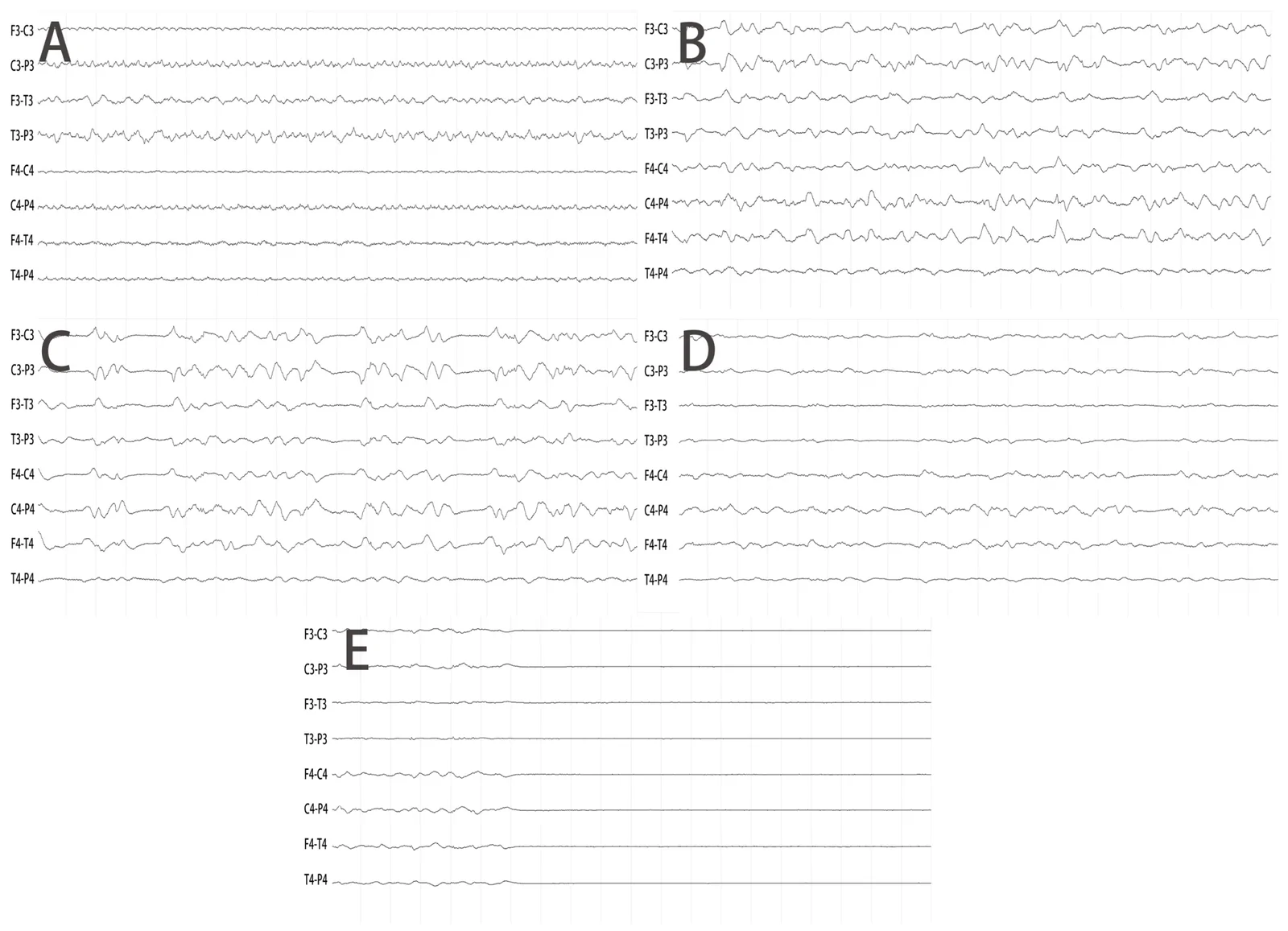
Electroencephalographic changes in experimental rabbits during stepwise steady-state graded intracranial hypertension. (**A**) Baseline; (**B**) ICP 15–20 mmHg; (**C**) ICP 25–30 mmHg; (**D**) ICP 35–40 mmHg; (**E**) terminal decompensation (END). In each panel the eight channels are, from top to bottom, F3-C3, C3-P3, F3-T3, T3-P3, F4-C4, C4-P4, F4-T4 and T4-P4.

Figure 5 presents 20 s segments of raw EEG at each of the five ICP levels and illustrates the progressive suppression of EEG activity as ICP increased. At baseline, the EEG showed continuous background activity with a regular α rhythm (Figure 5A); from 15–20 mmHg onward it slowed, with the background rhythm shifting from α toward θ (Figure 5B); at 25–30 mmHg it became dominated by slow activity, with near-disappearance of α and β (Figure 5C); at 35–40 mmHg diffuse high-amplitude slow waves emerged (Figure 5D); and at END it progressed to burst suppression and finally to a near-isoelectric pattern (Figure 5E).

#### 3.3.2. Quantitative Changes in Band Power

**Figure 6:**
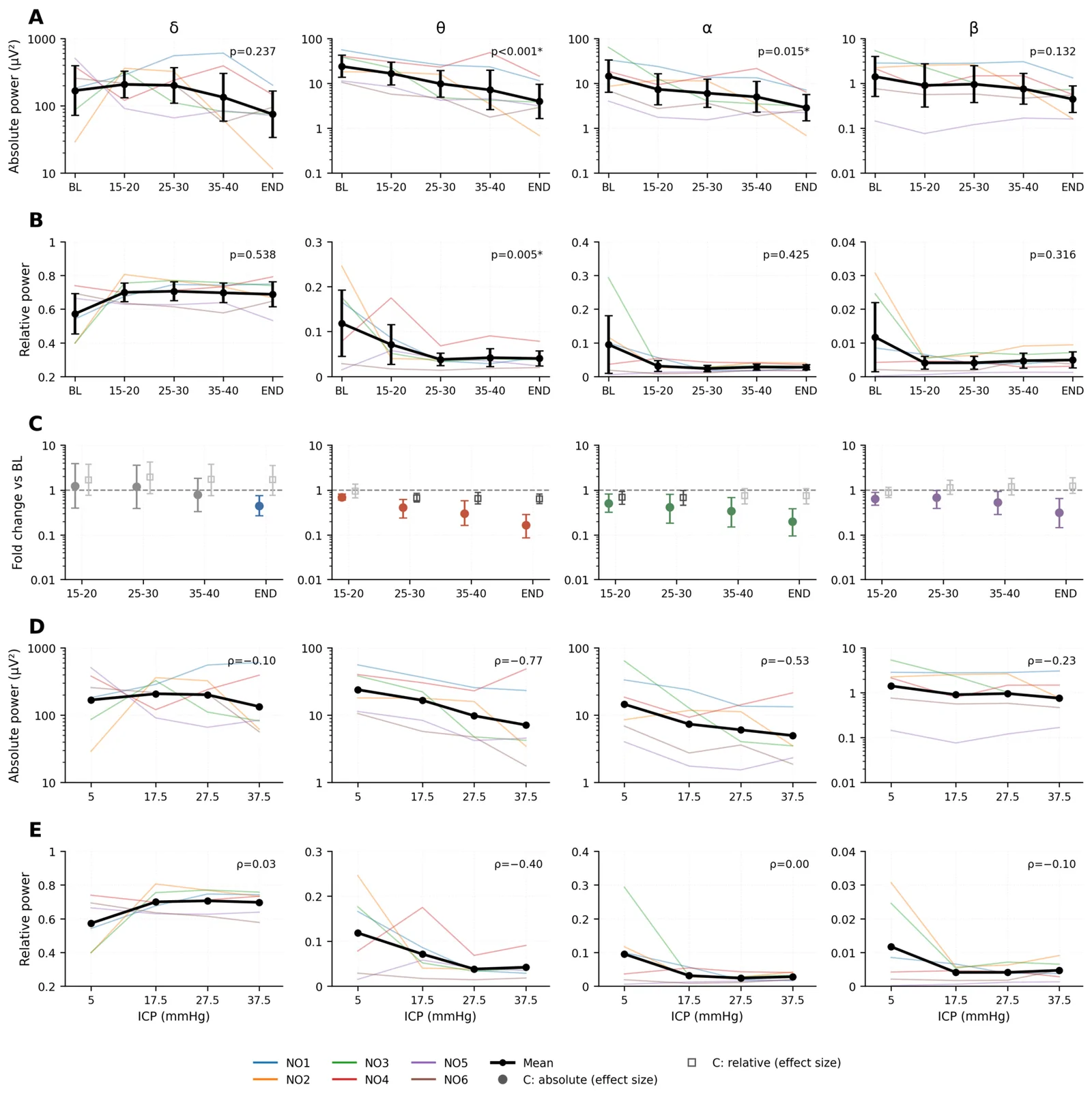
Changes in EEG power during graded intracranial hypertension (*n* = 6). (**A**) Absolute power (log scale); (**B**) relative power; (**C**) fold change relative to baseline (log scale; dashed line at unity (1.00)); (**D**,**E**) absolute and relative power against ICP (x-axis, interval midpoint; END excluded). Thin lines—individual rabbits; thick lines—mean ± 95% CI. *—FDR-corrected q < 0.05.

Figure 6A shows the absolute power of the four bands. Progressive suppression arose mainly from θ and α, which decreased continuously and monotonically as ICP rose (time-effect *p* < 0.001 for θ and *p* = 0.015 for α; the θ decline was also highly consistent across animals, Kendall W = 0.78), whereas δ and β showed no clear change (*p* = 0.237 and *p* = 0.132, respectively; in particular, δ did not increase).

Figure 6B shows each band’s relative share of total power. Owing to the attenuation of θ and α, the δ share rose passively from 58.51% at baseline to 70.40% at 15–20 mmHg and then plateaued; at END, the θ, α and β shares were only 4.3%, 3.2% and 0.6%. After CLR transformation of these compositional data (which sum to one), only the change in the θ share was significant (*p* = 0.005), indicating that the rising slow-wave share reflected a reciprocal change following the loss of fast activity rather than genuine strengthening of slow activity.

#### 3.3.3. Correlation Between the Magnitude of Band-Power Change and ICP

Figure 6C uses the fold change relative to baseline (unity = no change) to depict the magnitude of the decline: θ fell stepwise to 0.70, 0.41, 0.30 and 0.17 of baseline and α to 0.51, 0.42, 0.34 and 0.20, with confidence intervals that did not cross unity; δ fell below baseline only at END (0.45), whereas β varied in direction without significance. When power was plotted against ICP (Figure 6D,E), absolute θ power showed the strongest negative correlation (ρ = −0.77), followed by α (−0.53), with weaker associations for β and δ (−0.23 and −0.10); relative power showed consistently weaker associations. Given *n* = 6, pointwise comparisons after Holm correction had limited sensitivity (adjusted *p* = 0.130–1.000); overall trends and effect sizes were therefore used as the primary criteria.

### 3.4. Waveform Changes and Quantitative Analysis of the aEEG Under Graded Intracranial Hypertension

#### 3.4.1. Gradual Evolution of aEEG Waveforms

**Figure 7:**
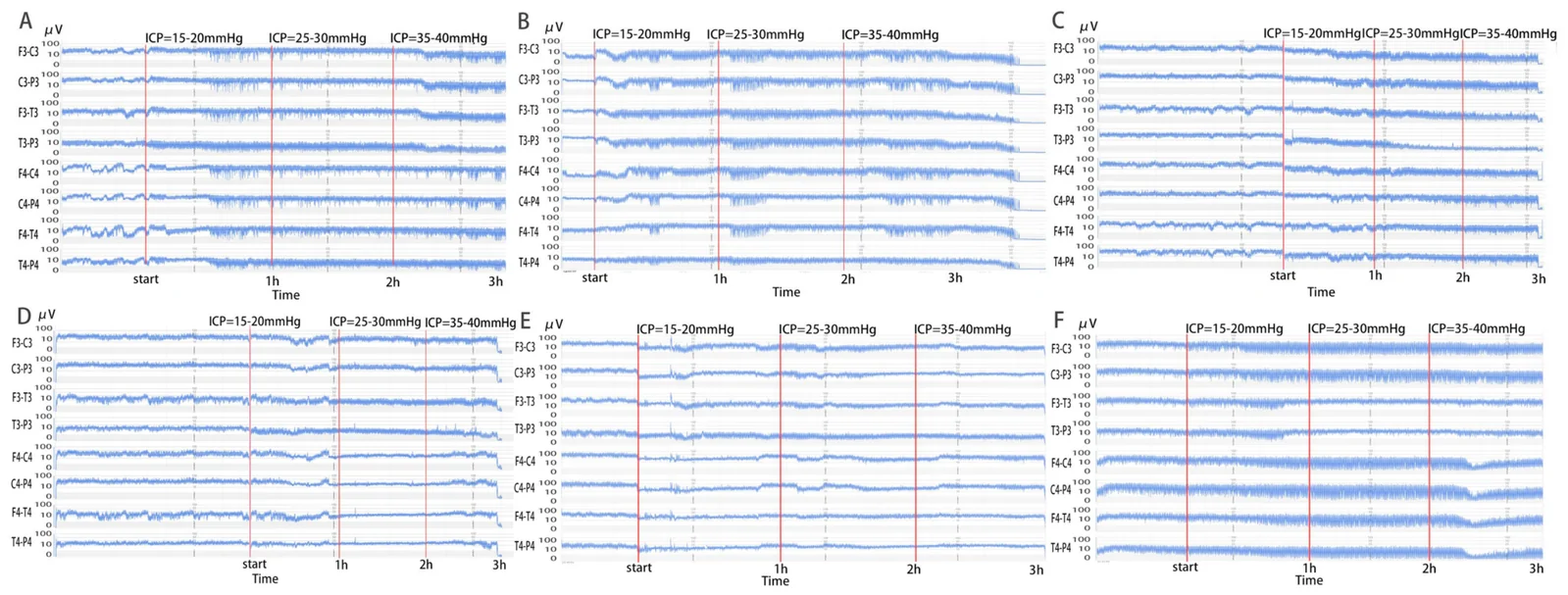
Continuous multichannel EEG recordings of the six experimental rabbits during graded intracranial hypertension. (**A**–**F**) correspond to rabbits 1–6 (top row (**A**–**C**), bottom row (**D**–**F**)); in each panel the eight EEG channels are shown from top to bottom (F3-C3, C3-P3, F3-T3, T3-P3, F4-C4, C4-P4, F4-T4, T4-P4); the y-axis is voltage (μV) and the x-axis is recording time (from start to about 3 h); red vertical lines delimit BL and the 15–20, 25–30 and 35–40 mmHg stages.

Figure 7 uses strong time compression to display the full trajectories of continuous eight-channel EEG in all six rabbits (A–F correspond to rabbits 1–6), with red vertical lines delimiting the ICP stages, allowing the prevalence and timing of suppression to be examined at the individual level. The direction of change was consistent across rabbits: EEG amplitude was progressively depressed from approximately 50–100 μV at baseline, slow activity increased at 25–30 mmHg, and all channels fell to 10–30 μV at 35–40 mmHg (rabbits 1 and 3 exhibited burst-suppression-like patterns); at END, rabbit 2 reached electrocerebral silence in all eight channels (lower margin < 0.4 μV), whereas the remaining animals approached similarly low amplitudes. Only the timing and degree differed between animals. This corroborates the power decline in Section 3.3 and indicates that EEG suppression is a gradual, reproducible process across animals.

#### 3.4.2. Gradual Changes and Trends of the Three aEEG Indices

**Figure 8:**
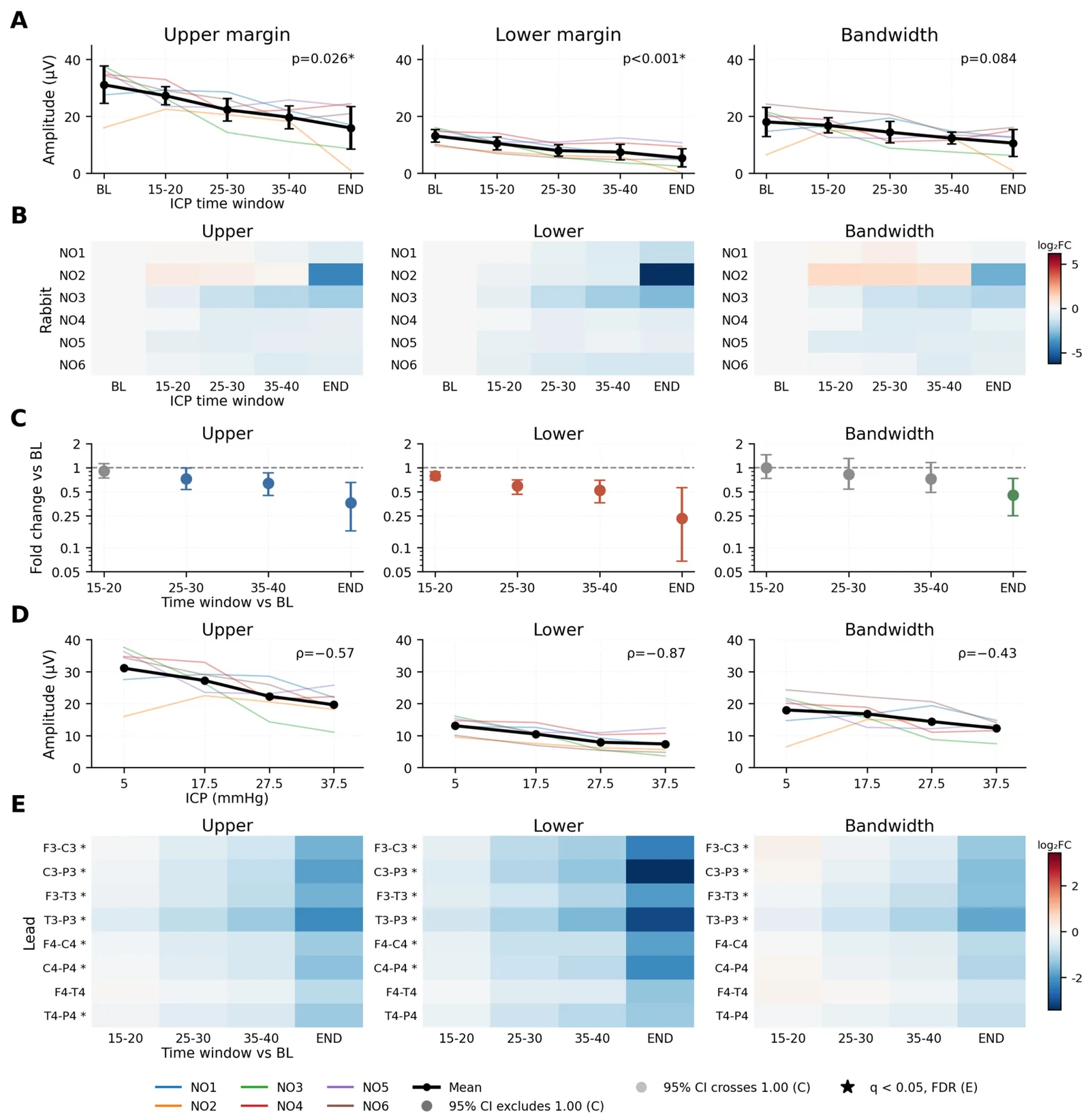
Quantitative analysis of the aEEG upper margin, lower margin and bandwidth during graded intracranial hypertension (*n* = 6). Columns are, in order, upper margin, lower margin and bandwidth. (**A**) Change across ICP windows (thick line—mean ± 95% CI; randomization *p* values within subplots); (**B**) heatmap of each rabbit’s log2 fold change relative to BL; (**C**) fold change relative to BL (dashed line at unity (1.00)); (**D**) correlation with ICP (END excluded); (**E**) channel-wise log2 fold-change heatmap; *—FDR-corrected q < 0.05. In rabbit 2 at END, the lower margin was <0.4 μV in all eight channels (electrocerebral silence) and was exactly zero in T4-P4; this channel–window value was therefore computed from the remaining five animals (*n* = 5), whereas *n* = 6 was used for all other channel–window values.

Figure 8A compares the aEEG upper margin (which reflects higher-amplitude activity), lower margin (which reflects the floor of background activity) and their difference (bandwidth) across the five windows (BL, 15–20, 25–30, 35–40 mmHg and END). The lower margin decreased progressively, monotonically and early, and remained suppressed across subsequent levels (Friedman *p* < 0.001; Page trend z = 3.68, *p* < 0.001; Kendall W = 0.86). The upper margin decreased overall (*p* = 0.026) but was no longer significant after END was excluded (*p* = 0.091), indicating that its decline occurred mainly at the terminal stage. Bandwidth showed no consistent directional change (*p* = 0.084; Kendall W = 0.33).

#### 3.4.3. Magnitude of aEEG Change, Correlation and Channel-Wise Differences

Figure 8C shows the magnitude of decline as the fold change relative to baseline: the lower margin fell stepwise to 0.79, 0.59, 0.52 and 0.23 (END) of baseline, with confidence intervals that did not cross unity, whereas the confidence interval of the upper margin crossed unity at all but the last three time points, and that of bandwidth crossed unity at all but END. When plotted against ICP (Figure 8D), the lower margin showed a strong negative correlation (ρ = −0.87), stronger than those of the upper margin (−0.57) and bandwidth (−0.43). The channel-wise heatmap (Figure 8E) showed that the change was widespread: after FDR correction, the lower margin was significant in six channels, the upper margin in seven and bandwidth in four (predominantly on the left side; channels are descriptive, with inference at the animal level); the lower-margin decline was most consistent across animals at F3-C3 and C3-P3 (Kendall W = 0.91 and 0.92). The aEEG lower margin thus declined early and markedly, was closely associated with ICP and was reproducible across channels; the upper margin changed late and bandwidth showed low consistency, limiting their value. The selection stability and out-of-sample performance of the lower margin as a candidate feature are examined in Section 3.6.

### 3.5. Relationship Between qEEG and Cerebral Perfusion Pressure

**Figure 9:**
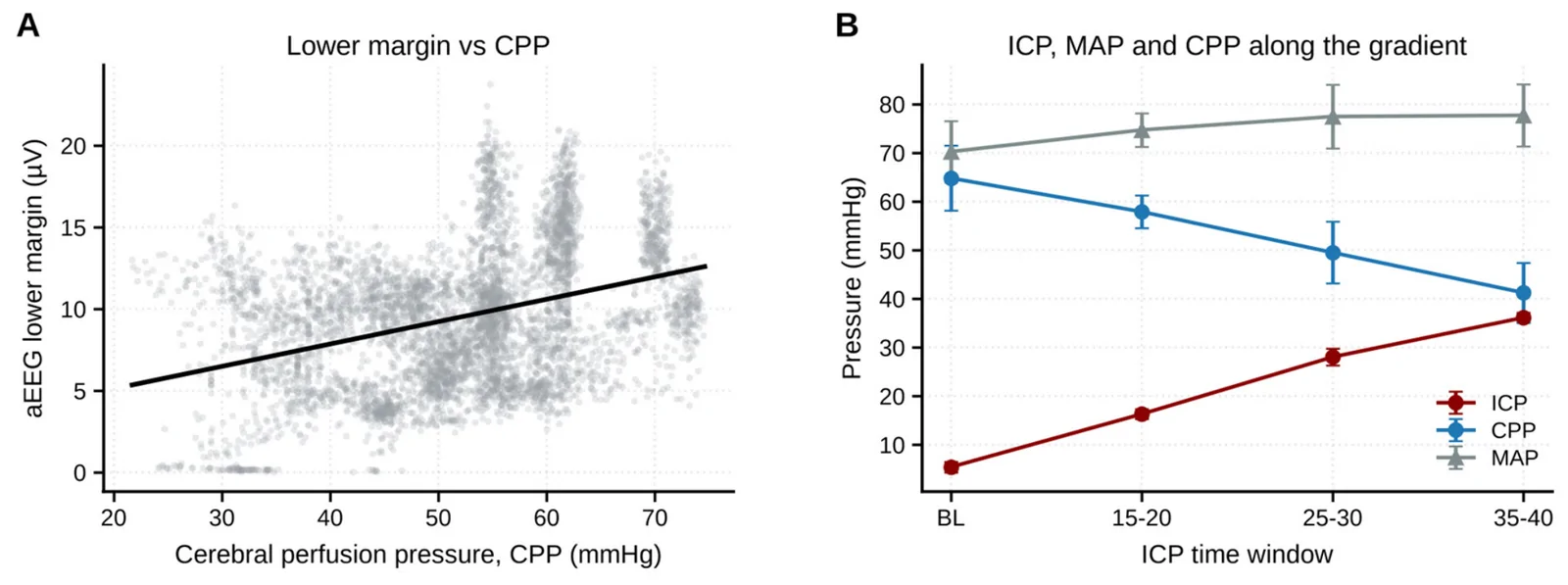
Relationship between qEEG and cerebral perfusion pressure (CPP; *n* = 6). (**A**) Time-point aEEG lower margin (mean across channels) versus CPP across all rabbits (semi-transparent points; black line—linear fit); the animal mean Spearman r_s over the four windows was +0.87 (positive in 6/6 rabbits). (**B**) Group mean ICP, CPP and MAP across the four windows (error bars, SD): MAP rose actively through autoregulation and the Cushing response (+7.47 mmHg, with heart-rate slowing), but the rise was only about one quarter of the ICP rise, so CPP fell from 64.78 to 41.24 mmHg; relative to the rabbit lower limit of autoregulation (~50 mmHg) and Cushing point (~42 mmHg) [10], the animals remained above the lower limit at 15–20 mmHg and approached it only at 25–30 mmHg.

CPP is defined as MAP−ICP. As ICP rose, MAP actively compensated through autoregulation and the Cushing response (70.23→77.70 mmHg, with heart-rate slowing, ρ = −0.75), but its +7.47 mmHg rise was only about one quarter of the ICP rise (+30.72; −3% to 46% across animals). CPP therefore fell from 64.78 to 41.24 mmHg (Figure 9B). In a rabbit model of experimental intracranial hypertension, the lower limit of autoregulation (LLA) was approximately 50 mmHg CPP and the Cushing point approximately 42 mmHg [10], rather than the ~60 mmHg obtained historically by lowering arterial pressure in the normal brain. Against this reference, the animals remained above the LLA in the 15–20 mmHg window (57.88 mmHg, 0/6 below 50), the group mean approached the LLA in the 25–30 mmHg window (49.49, 3/6 below 50), and it neared the Cushing point in the 35–40 mmHg window (41.24, 3/6 below 45); these thresholds are individual- and state-dependent and were used only as a reference because cerebral blood flow was not measured. The aEEG lower margin showed the strongest positive association with CPP (animal mean r_s = +0.87, positive in 6/6; Figure 9A), followed by θ (+0.77) and α (+0.53), which were near-mirror images of the correlations with ICP. Because ICP and CPP were almost perfectly coupled within animals (r = −0.95; variance inflation factor ≈ 11), their independent effects could not be separated reliably: a parallel within-animal regression of the lower margin on ICP and CPP (rabbit-indicator fixed effects) indicated that the ICP coefficient remained significant after adjustment for CPP (β = −0.35, 95% CI −0.56 to −0.14, *p* = 0.003), whereas the CPP coefficient after adjustment for ICP did not (β = −0.22, 95% CI −0.48 to 0.05, *p* = 0.11); the corresponding partial correlations were r = −0.66 and −0.39. Thus the qEEG changes, including the early lower-margin decline that began in the 15–20 mmHg window while CPP was still above the LLA, cannot be attributed uniquely to either elevated ICP or hypoperfusion; disentangling the two would require independent manipulation of MAP with simultaneous cerebral blood flow measurement.

### 3.6. Stability of Candidate-Feature Selection and Out-of-Sample Validation

**Figure 10:**
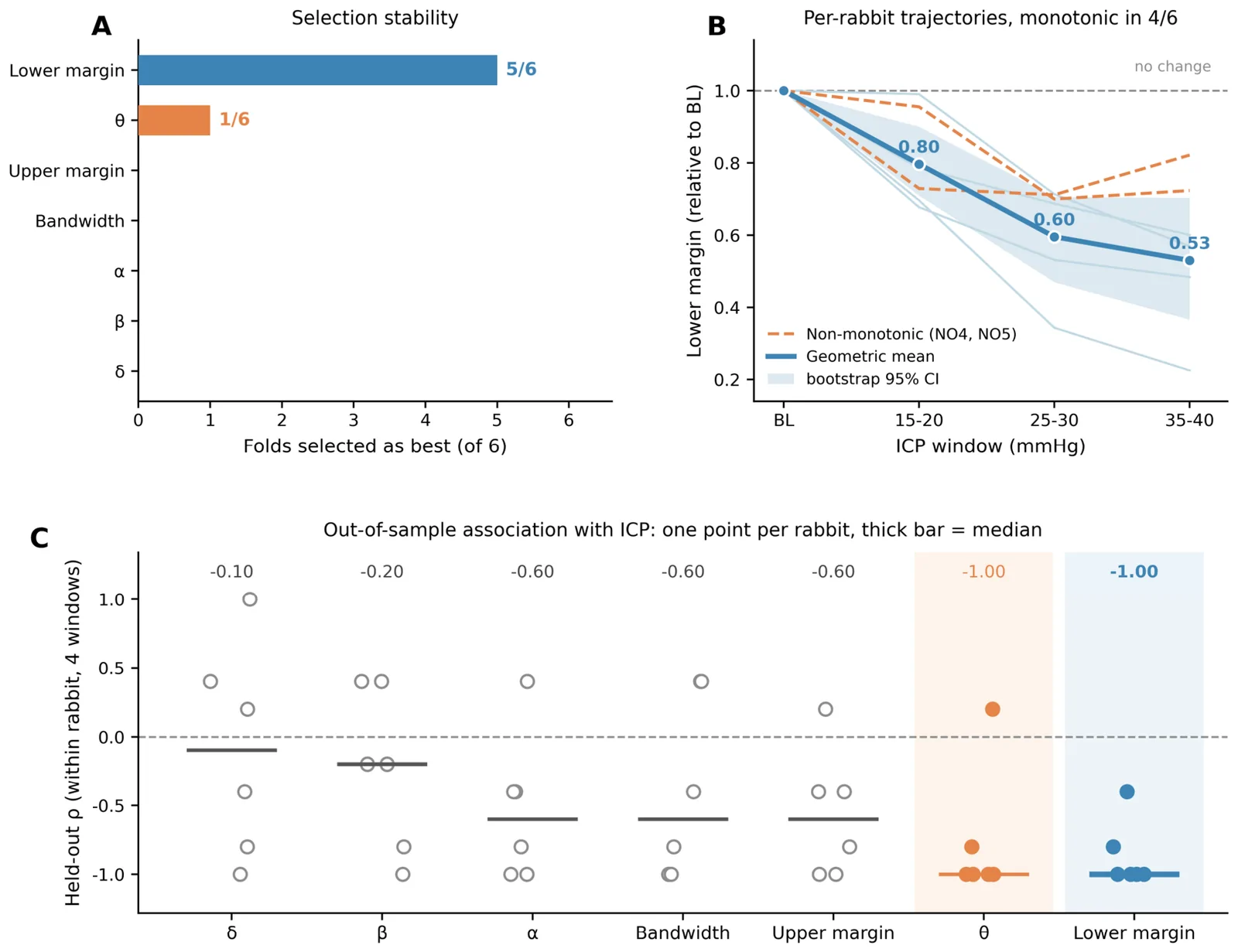
Stability of candidate-feature selection and leave-one-rabbit-out cross-validation (*n* = 6). (**A**) Number of folds in which each candidate was selected as optimal across the six LORO folds; (**B**) evolution of each rabbit’s aEEG lower margin relative to baseline (thin lines—individuals; thick line—geometric mean; light band, bootstrap 95% CI; orange dashed lines—the non-monotonic NO4 and NO5); (**C**) out-of-sample ρ of each candidate within held-out rabbits (one point per rabbit; horizontal line, median). With only four windows per rabbit and a ρ granularity of 0.2, the median and dispersion are used for interpretation. The δ, θ, α and β labels denote band absolute powers, and the upper-margin, lower-margin and bandwidth labels denote the aEEG indices.

Across the six LORO folds, the lower margin was selected in five of them and absolute θ power in the remaining one (Figure 10A), indicating that feature selection did not depend on any single individual. In the held-out rabbits, the lower margin decreased monotonically with ICP in 4/6, with a median out-of-sample ρ of −1.00 (range −1.00 to −0.40; Figure 10C) and smaller dispersion than θ (−1.00 to +0.20). NO4 and NO5 rebounded slightly at the 35–40 mmHg level (Figure 10B), the only two non-monotonic cases. Because each held-out ρ is computed from only four windows and therefore takes discrete values in increments of 0.2, the median of −1.00 reflects perfect monotonicity rather than a precise effect magnitude and should not be over-interpreted. With only six animals (five used for selection in each fold), the five-of-six agreement is not demonstrably superior to complete agreement and indicates internal consistency within this sample, but it does not establish a stable or generalizable selection rule; the analysis is best regarded as hypothesis generating rather than confirmatory.

## 4. Discussion

Using small, repeated balloon inflations, we established a stepwise, steady-state model of graded intracranial hypertension within the same animals and synchronously compared band power with the aEEG upper and lower margins and bandwidth. The principal findings were as follows: highly consistent pressure elevation (Kendall W = 0.96); progressive declines in absolute θ and α power without any increase in δ absolute power; and an early, individually consistent downward shift of the aEEG lower margin (W = 0.86; Spearman ρ = −0.87 with ICP), whereas the upper-margin decline was driven mainly by the end stage and bandwidth varied in direction. By decomposing “EEG suppression” into comparable band and aEEG components and tracing their continuous changes along the ICP gradient, we identified the aEEG lower margin as the prioritized candidate early-warning feature according to three prespecified criteria.

### 4.1. A Stepwise Steady-State Design Separating Pressure Effects from Inflation Speed, Individual Differences and Physiological Fluctuation

In clinical practice, ICP often rises paroxysmally and fluctuates, confounded by the primary disease and inter-individual differences, so that a pure pressure effect is difficult to isolate [13]. Existing animal models mostly inflate a balloon once and rapidly, with marked pressure overshoot [11,12]; Ciarrocchi et al. [14] related the φ angle derived from the power-spectrum slope to ICP across 11 episodes in three pigs (r = −0.85), but episode onset and offset were uncontrolled and comparison was confined to the two extremes of “normal” and “cerebral circulatory arrest”. By injecting only 0.1–0.5 mL at a time, allowing ICP to return to a new plateau within about 1 min after each brief jump and maintaining each of the three levels for about 1 h, we separated pressure level from inflation speed and individual baseline; it was precisely the retention of continuous intermediate gradients that revealed the temporal dissociation whereby “the lower margin moves first and the upper margin only at the very end”—comparing only the two extremes would compress this into an apparently synchronous global decline. Attributing the EEG changes to pressure itself also required ruling out respiratory and circulatory fluctuations: the stage effects of Pmean, PEEP and EtCO_2_ were small (η^2^ = 0.079, 0.023, 0.010) and their correlations with ICP were close to zero, with no significant independent effect after adjustment (together accounting for only 7.4% of variance); although MAP and HR shifted with the gradient, they followed circulatory compensation after the fall in CPP and were consequences rather than causes.

### 4.2. Band Power: θ and α Suppression Is a Shared Electrophysiological Phenotype of Hypoperfusion, While the “δ Rise” Is Relative Redistribution

As CPP fell along the gradient, neurovascular coupling and cerebral autoregulation were compromised [15], and the thalamocortical synchrony that maintains fast rhythms was suppressed early. The sensitivity of α activity to ischemia has been documented in cerebral infarction after subarachnoid hemorrhage [16], in ischemia during carotid-endarterectomy clamping [17], in hypoperfusion in hyper-acute ischemic stroke [18] and in the prediction of delayed cerebral ischemia [19,20]. In a model without primary vascular occlusion—pure mechanical graded compression—we reproduced progressive declines in absolute θ and α power (ρ = −0.77 and −0.53, respectively), suggesting that this decline is not specific to ischemic stroke but represents a shared downstream phenotype of cortical dysfunction under pressure loading and falling perfusion.

The slow waves warrant particular scrutiny. The relative δ share rose from 58.51% to 70.40%, superficially resembling the classic “EEG slowing”, yet its absolute power showed no significant trend (*p* = 0.237; ρ = −0.10), and CLR analysis found significance only for θ; the rising δ share therefore arose mainly from compositional redistribution after the generalized decline of θ, α and β (a “denominator effect”). Many qEEG indices are built on relative ratios and may, in progressive intracranial hypertension, misinterpret “loss of fast activity” as “increase in slow activity”. By cross-validating absolute power, relative share and CLR, we recommend using absolute power as the primary criterion.

### 4.3. Decoupling the Three aEEG Components: The Lower Margin Is the Priority Order Parameter, Whereas “Bandwidth Narrowing” Is Unreliable

aEEG has been used mainly to assess neonatal brain maturation [21,22] and prognosis after adult cardiac arrest [23], with limited evidence outside neonates [24] and mostly case reports in cerebral herniation [25,26]. By decoupling aEEG into its upper margin, lower margin and bandwidth within the same animals, we found that the three components were not synchronous: the lower margin represents the “floor” of background activity and shifted down monotonically with ICP (W = 0.86; Page trend z = 3.68, *p* < 0.001; ρ = −0.87), with 95% CIs that did not cross the no-change line at any time point; the upper margin represents the “ceiling” supported by residual bursts and was no longer significant after END was excluded (*p* = 0.091), i.e., a terminal event; bandwidth showed low directional consistency (*p* = 0.084; W = 0.33). Absolute θ power was slightly inferior in both individual consistency (W = 0.78) and ICP correlation (ρ = −0.77) and additionally requires frequency decomposition, whereas the lower margin is read continuously as a single value and is therefore better suited to bedside monitoring and providing alerts and alarms.

This dissociation is mechanistically plausible: the lower margin corresponds to the depth of suppression and responds early to falling CPP, whereas the upper margin collapses only when suppression becomes widespread near herniation; bandwidth is their difference, so an early downward shift of the lower margin should widen it, whereas the terminal collapse of the upper margin narrows it, and these opposing forces render its direction indeterminate. This mechanism is consistent with the evolution of CPP: the lower margin began to shift downward in the 15–20 mmHg window while CPP was still above the rabbit lower limit of autoregulation (~50 mmHg), and CPP approached that limit only at 25–30 mmHg and the Cushing point (~42 mmHg) at 35–40 mmHg; the lower margin nonetheless correlated strongly with CPP (r_s = +0.87). Because ICP and CPP were tightly coupled and could not be separated, the early decline cannot be assigned uniquely to either the direct pressure load or perfusion decompensation, although cortical background activity is highly sensitive to hypoperfusion. Hence, in acquired intracranial hypertension of the mature brain, equating “bandwidth narrowing” with deterioration is unreliable. Spatially, the channels with the most consistent lower margin—F3-C3 and C3-P3 (W = 0.91 and 0.92)—and those with significant bandwidth were ipsilateral to the balloon (left side), suggesting a focal-to-global gradient of suppression.

From a warning perspective, beyond the three prespecified screening criteria, a candidate feature worth advancing to validation should also meet four operational conditions: it should precede deterioration of clinical signs, vary monotonically with pressure, show a consistent direction across individuals, and output a single value amenable to automatic alarms. Of the seven candidates, the aEEG lower margin was the only one to satisfy all of them: it began to fall at the early 15–20 mmHg gradient, its CI did not cross the no-change line, it was strongly and negatively correlated with ICP (ρ = −0.87), and it is output continuously without frequency decomposition, whereas θ and α require frequency decomposition, the upper margin is late and bandwidth is inconsistent. At END, all eight lower-margin channels of rabbit 2 were below 0.4 μV, near electrocerebral silence [23,27], while circulation had not yet collapsed, indicating that EEG suppression can precede vital-sign decompensation. We emphasize that satisfying these four conditions indicates only that the lower margin deserves prioritized validation, not that its early-warning performance is established; before clinical use, an alarm threshold must be fixed in advance and its sensitivity, specificity, false-alarm rate and lead time estimated in independent cohorts.

### 4.4. Logic of Evidence Under a Small Sample

At *n* = 6, pairwise comparisons were non-significant after Holm correction; however, at *n* = 6 the smallest attainable two-sided *p* for a paired Wilcoxon test is 0.031, so statistical power is inherently limited. We therefore did not base conclusions on isolated *p* values, but cross-corroborated them with permutation tests, Page’s trend test, bootstrap 95% CIs, Kendall W and ρ; θ, α and the lower margin were concordant in direction across all lines of evidence. This convergence notwithstanding, the study is exploratory and the evidence is preliminary. In particular, each LORO fold selected features from only five animals and the held-out set comprised a single rabbit; with only six animals, selection in 5/6 folds is not a meaningfully different outcome from 6/6 and therefore shows only relative robustness within this sample rather than a stable population-level estimate. The median out-of-sample ρ of −1.00 likewise rests on six held-out animals; because each animal contributes only four windows, a four-point Spearman ρ has very few achievable values (granularity 0.2), so −1.00 is close to one of the only possible values and may reflect this discretization rather than a genuinely perfect monotonic relationship and its magnitude should not be over-interpreted. We accordingly frame the lower margin as a prioritized candidate for further testing rather than a validated marker and avoid claims of sensitivity or specificity that such a small sample cannot support.

### 4.5. Limitations and Future Directions

Several limitations remain. First, because MAP was not independently manipulated and cerebral blood flow was not measured, ICP elevation and CPP decline were almost perfectly coupled and could not be separated; although the lower-margin association with ICP remained after controlling for CPP, the attribution of the EEG changes remains inferential. Second, the experiments used healthy single-sex rabbits under anesthesia without a sham-operated group, so the effects of anesthesia and cumulative time—which co-varied with ICP—cannot be excluded. Third, this was an exploratory screening with internal validation only: the sample was small (*n* = 6; each fold selected from only five animals), an independent cohort and fixed thresholds were lacking, and sensitivity, specificity and false-alarm rate were not determined. Future work should enlarge the sample, independently manipulate MAP while measuring cerebral blood flow and CPP simultaneously, and validate the feature in an independent cohort.

## 5. Conclusions

In this exploratory study, pressure-controlled graded intracranial hypertension was reproduced in a small rabbit cohort (*n* = 6). qEEG indices evolved asynchronously along the gradient: θ and α power declined progressively, the aEEG lower margin shifted downward early and was selected in 5/6 LORO folds (the median out-of-sample ρ of −1.00 reflects the coarse discretization of four windows rather than proven perfect monotonicity), and the upper margin collapsed only terminally. MAP compensation was insufficient and CPP fell from 64.78 to 41.24 mmHg, approaching the rabbit lower limit of autoregulation (~50 mmHg) and the Cushing point (~42 mmHg) at the later windows. However, because ICP and CPP were tightly coupled and could not be separated, the early lower-margin decline cannot be attributed uniquely to either elevated ICP or hypoperfusion. The lower margin is therefore a candidate rather than a confirmed early-warning feature, and it and its threshold remain to be confirmed in larger independent cohorts with simultaneous measurement of MAP, CPP and cerebral blood flow.

## Figures and Tables

**Table 1:** Baseline physiological parameters of the experimental rabbits (*n* = 6, mean ± SD).

Parameter	Mean ± SD	CV (%)
Age (months)	3.50 ± 0.00	—
Body weight (kg)	2.49 ± 0.05	1.97
Body temperature (°C)	38.48 ± 0.08	0.20
Heart rate (beats/min)	282.17 ± 18.10	6.41
ICP (mmHg)	5.43 ± 1.09	20.07
MAP (mmHg)	70.34 ± 6.08	8.64
CPP (mmHg)	64.95 ± 6.58	10.12
Respiratory rate (breaths/min)	37.69 ± 1.49	3.95
Tidal volume VT (mL)	20.51 ± 1.07	5.23
EtCO_2_ (mmHg)	37.10 ± 0.69	1.85
Mean airway pressure Pmean (cmH_2_O)	7.25 ± 0.40	5.52
PEEP (cmH_2_O)	3.00 ± 0.02	0.52
Spectral entropy	51.47 ± 1.55	3.02
Compliance (mL/cmH_2_O)	4.85 ± 0.52	10.69

Note: ICP—intracranial pressure; MAP—mean arterial pressure; CPP—cerebral perfusion pressure (CPP = MAP − ICP); EtCO_2_—end-tidal carbon dioxide; Pmean—mean airway pressure; VT—tidal volume; PEEP—positive end-expiratory pressure; CV—coefficient of variation. Each parameter was averaged over the interval after vital signs stabilized and before the first inflation.

## Data Availability

The de-identified experimental data that support the findings of this study, together with the code for EEG segmentation, spectral and aEEG quantification, candidate feature selection and leave-one-rabbit-out cross-validation, are available from the corresponding author upon reasonable request, to permit verification of the selection process and out-of-sample estimates. This study did not involve public clinical registration or participant-level private data.

## Supplementary Materials

The following supporting information can be downloaded at https://www.mdpi.com/article/10.3390/brainsci16101116/s1, Supplementary Material S1: ARRIVE 2.0 Author Checklist—Full Version.
