# Supplementary material for: Dynamic Evolution of Quantitative Electroencephalography During Graded Intracranial Hypertension and Screening of Candidate Early-Warning Features in a Rabbit Model
Source: Brain Sci. 2026 Oct 16;16(10):1116. doi: 10.3390/brainsci16101116 (PMC13702719; doi:10.3390/brainsci16101116)
Supplement: Supplementary file 1 [file brainsci-16-01116-s001.zip › brainsci-4598378-supplementary.zip!/brainsci-4598378-supplementary/Supplementary Material S1.pdf]

ARRIVE 2.0 Author Checklist — Full Version

Manuscript: Dynamic Evolution of Quantitative Electroencephalography during Graded Intracranial Hypertension and Screening of Candidate Early-Warning Features in a Rabbit Model (Brain Sciences)

IACUC protocol: IACUC FJMU 2022-0641 (approved 9 June 2022). Section/figure/table numbers refer to the English manuscript.

A. Essential 10 (Items 1–10)

Item	Recommendation	Reported in this study	Location (§/Fig./Table)
**1. Study design**	For each experiment, state the study design, including control/experimental groups, type of control and any steps taken to minimise bias (randomisation, blinding).	Single-group, longitudinal, within-animal repeated-measures animal study; each animal served as its own baseline control (no separate control group). Stage order was deliberately non-randomized because ICP had to rise stepwise; balloon inflation and EEG acquisition were separated from data analysis, and the statistical analysis was performed in a blinded manner by an investigator not involved in the procedures.	§2.1; §4.5; Fig. 2
**2. Sample size**	(a) Specify the exact number of animals in each group and the number of independent units/ repeats; (b) state how the sample size was derived.	(a) Eight male New Zealand rabbits entered; two were excluded and six completed (n=6 independent animals; eight EEG channels were within-animal repeats). (b) n=6 was set with reference to comparable large-animal studies; a formal a priori power calculation was not performed, and the resulting limited power is acknowledged and addressed by convergent non-parametric evidence.	§2.1; §3.1; §4.4; Table 1
**3. Inclusion / exclusion criteria**	(a) State pre-specified inclusion/exclusion criteria; (b) describe whether criteria were set a priori.	Criteria were fixed before analysis. Excluded: one animal with circulatory failure during catheterization and one reaching a humane endpoint (refractory status epilepticus); both were excluded before analysis; six completed all five ICP windows.	§2.1
**4. Randomization**	(a) State whether and how animals were randomized to groups/order; (b) describe treatment allocation.	Not applicable to treatment groups (single group, self-baseline). The order of ICP stages was not randomized because ICP had to increase stepwise; this is stated explicitly as a design constraint.	§2.1; §4.5
**5. Blinding**	(a) State who was blinded to group/treatment; (b) describe the blinding procedure.	The investigators performing balloon inflation and EEG acquisition were separated from data analysis; an investigator not involved in the	§2.1

		procedures performed the statistical analysis blinded to animal identity and ICP stage where feasible.	
**6. Outcome measures**	(a) Define primary/secondary outcomes; (b) state how and when they were measured.	Seven prespecified candidate features (absolute and relative power of the δ, θ, α and β bands; aEEG upper margin, lower margin, bandwidth) and three selection criteria (evolution timing, individual consistency quantified by Kendall W, and strength of ICP association quantified by Spearman ρ) were fixed before analysis; definitions and computation are given.	§2.4; §2.5; Figs. 6, 8
**7. Statistical methods**	(a) Provide full statistical methods; (b) name software and version.	Animal-level non-parametric inference (n=6); Friedman with Kendall W; Wilcoxon signed-rank with Holm correction; Page trend test; CLR transformation for relative power; rabbit-clustered bootstrap 95% CIs; Spearman correlation; fixed-effects models with semipartial R^2; FDR for channel-level descriptive tests; two-sided $\alpha=0.05$. Software: Python 3.8.	§2.5
**8. Experimental animals**	(a) Species/strain/sex/age/weight/source; (b) housing, husbandry, welfare-related details.	Conventional-grade male New Zealand rabbits, 3.5 months, 2.42–2.55 kg, from Songlian Experimental Animal Farm (SCXK (Hu) 2022-0006). After arrival the rabbits underwent a 7-day acclimatization/quarantine period before any procedure; each was singly housed in a standard stainless-steel rabbit cage (perforated floor with foot-rest, resting platform) with corn-cob bedding and an autoclaved wooden chew block as enrichment, at 22–25 °C and 40–70% relative humidity under a 12 h light/dark cycle, fed standard rabbit maintenance pellets with ad libitum water.	§2.1; IRB Statement
**9. Experimental procedures**	(a) Describe procedures with sufficient detail (anesthesia, analgesia, monitoring, model, acquisition); (b) state any protocol modifications.	Detailed: isoflurane induction + ethyl carbamate anesthesia titrated to spectral entropy 40–60; tracheostomy/mechanical ventilation; femoral arterial/venous lines; ICP probe and eight-channel bipolar EEG placement and settings; stepwise left-parenchymal water-balloon inflation; EEG/aEEG	§2.2–2.4; Figs. 1, 2

		processing pipeline. No unplanned protocol modification.	
**10. Results**	(a) Report number of animals included in each analysis; (b) use absolute numbers and effect sizes, not only p values.	All analyses used n=6, except a single channel–window aEEG value (T4-P4 at END in rabbit 2, which measured zero), which was computed from the remaining five animals (n=5) and is explicitly noted in the Figure 8 legend. Means ± SD, CV, η^2, Kendall W, Spearman ρ, fold changes with 95% CIs and exact p values are reported.	§3.1–3.5; Table 1; Figs. 3–9

B. Recommended Set (Items 11–21)

Item	Recommendation	Reported in this study	Location (§/Fig./Table)
**11. Abstract**	Provide a structured abstract with background, objectives, methods, results, conclusions.	Structured abstract (Background and Objectives; Materials and Methods; Results; Conclusions), ~250 words.	Abstract
**12. Background**	Give scientific background, rationale and how the study advances the field.	Gap (confounded clinical data; two-extreme animal models; single-index studies) and rationale are stated, with key literature.	§1
**13. Objectives**	State objectives/aims/hypotheses, primary and secondary.	Central question and aims stated; seven candidates and three prespecified criteria constitute the testable objective.	§1 (end); §2.1
**14. Ethical statement**	Ethics committee, approval, related licenses/permits; humane endpoints and euthanasia.	IACUC of Fujian Medical University, IACUC FJMU 2022-0641, approved 9 June 2022; ARRIVE 2.0; breeder license SCXK (Hu) 2022-0006; predefined humane endpoints and euthanasia (ethyl carbamate then 10% KCl under deep anesthesia).	IRB Statement; §2.1
**15. Housing and husbandry**	Housing conditions, acclimatization, welfare assessment before experiments.	7-day acclimatization/quarantine after arrival; single housing in standard stainless-steel rabbit cages with resting platform, corn-cob bedding and chew-block enrichment; 22–25 °C, 40–70% humidity, 12 h light/dark, standard pellets and ad libitum water; general health/appetite/behavior checked daily.	§2.1
**16. Animal care & monitoring**	Post-procedural care, monitoring frequency/parameters, humane endpoint criteria and actions.	Depth of anesthesia monitored (corneal reflex; spectral entropy 40–60); continuous multi-parameter monitoring; explicit humane endpoints and euthanasia procedure.	§2.1; §2.2
**17. Interpretation /**	Interpret results in light of	Mechanistic interpretation,	§4.1–4.5

**implications**	objectives/hypotheses; distinguish scientific effects from biological variation; comment on limitations.	denominator-effect clarification, aEEG-component decoupling, and explicit limitations.	
**18. Generalizability / translation**	Discuss species/model limits and translation to humans.	Single-sex healthy anesthetized animals; mechanical lesion; no cerebral blood-flow measurement; no independent cohort—discussed as translation limits.	§4.5
**19. Protocol registration**	State whether a protocol was registered and where it can be accessed.	Not applicable; no formal preregistration (animal study). The analysis plan and candidate features were nevertheless fixed before analysis.	§2.1
**20. Data access**	State where data/code supporting results can be found.	De-identified data and analysis code available from the corresponding author upon reasonable request.	Data Availability Statement
**21. Declaration of interests**	Declare conflicts of interest and funding sources.	No conflicts of interest; funded by the Natural Science Foundation of Fujian Province (2021J01368).	Conflicts of Interest; Funding
