# Supplementary material for: Dynamic Evolution of Quantitative Electroencephalography During Graded Intracranial Hypertension and Screening of Candidate Early-Warning Features in a Rabbit Model
Source: Brain Sci. 2026 Oct 16;16(10):1116. doi: 10.3390/brainsci16101116 (PMC13702719; doi:10.3390/brainsci16101116)
Supplement: Supplementary file 1 [file brainsci-16-01116-s001.zip › brainsci-4598378-tex.zip!/Definitions/brainsci-4598378-g002.pdf]

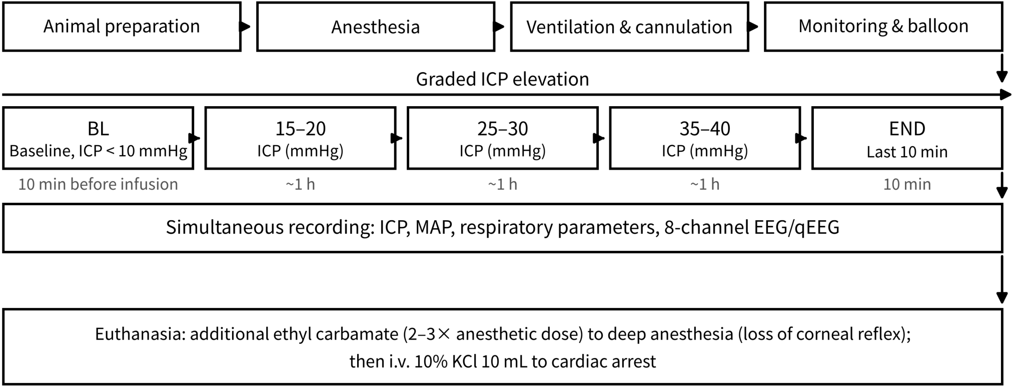

Animal preparation
Anesthesia
Ventilation & cannulation
Monitoring & balloon
Graded ICP elevation
BL
Baseline, ICP < 10 mmHg
10 min before infusion
15–20
ICP (mmHg)
~1 h
25–30
ICP (mmHg)
~1 h
35–40
ICP (mmHg)
~1 h
END
Last 10 min
10 min
Simultaneous recording: ICP, MAP, respiratory parameters, 8-channel EEG/qEEG
Euthanasia: additional ethyl carbamate (2–3× anesthetic dose) to deep anesthesia (loss of corneal reflex);
then i.v. 10% KCl 10 mL to cardiac arrest
