# Supplementary material for: Dynamic Evolution of Quantitative Electroencephalography During Graded Intracranial Hypertension and Screening of Candidate Early-Warning Features in a Rabbit Model
Source: Brain Sci. 2026 Oct 16;16(10):1116. doi: 10.3390/brainsci16101116 (PMC13702719; doi:10.3390/brainsci16101116)
Supplement: Supplementary file 1 [file brainsci-16-01116-s001.zip › brainsci-4598378-tex.zip!/Definitions/brainsci-4598378-g003.pdf]

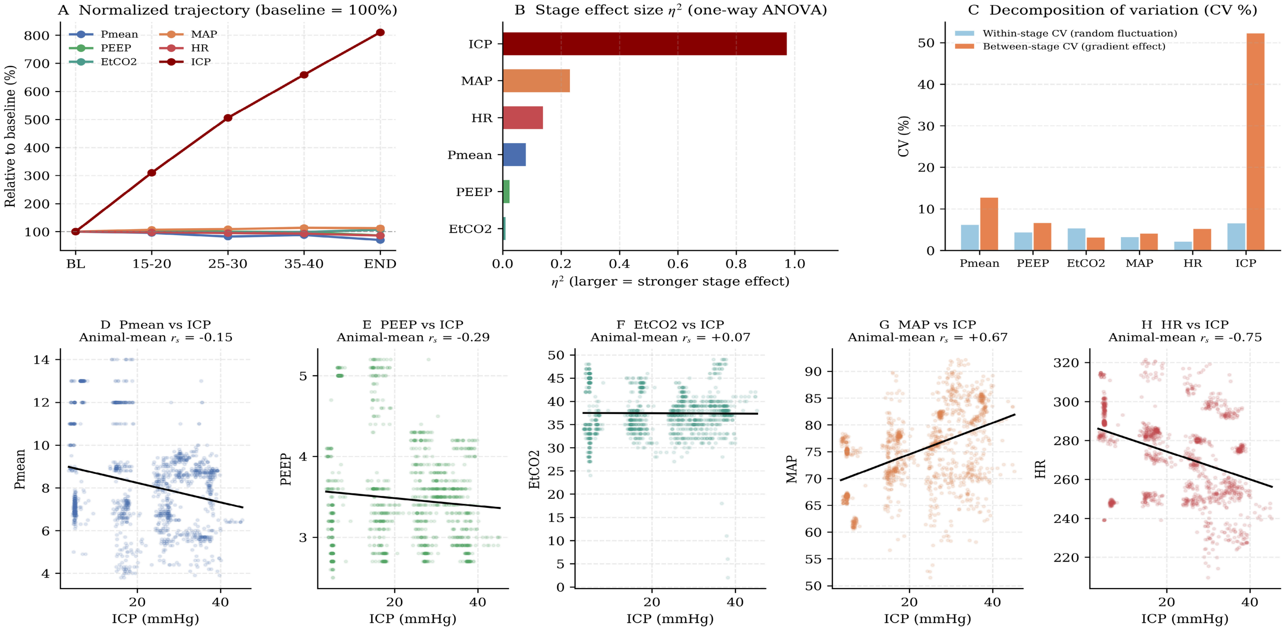

A Normalized trajectory (baseline = 100%)
Pmean
PEEP
EtCO2
MAP
HR
ICP
Relative to baseline (%)
BL
15-20
25-30
35-40
END
B Stage effect size η^2 (one-way ANOVA)
η^2 (larger = stronger stage effect)
C Decomposition of variation (CV %)
Within-stage CV (random fluctuation)
Between-stage CV (gradient effect)
CV (%)
D Pmean vs ICP
Animal-mean r_s = -0.15
E PEEP vs ICP
Animal-mean r_s = -0.29
F EtCO2 vs ICP
Animal-mean r_s = +0.07
G MAP vs ICP
Animal-mean r_s = +0.67
H HR vs ICP
Animal-mean r_s = -0.75
ICP (mmHg)
