# Supplementary material for: Dynamic Evolution of Quantitative Electroencephalography During Graded Intracranial Hypertension and Screening of Candidate Early-Warning Features in a Rabbit Model
Source: Brain Sci. 2026 Oct 16;16(10):1116. doi: 10.3390/brainsci16101116 (PMC13702719; doi:10.3390/brainsci16101116)
Supplement: Supplementary file 1 [file brainsci-16-01116-s001.zip › brainsci-4598378-tex.zip!/Definitions/brainsci-4598378-g004.pdf]

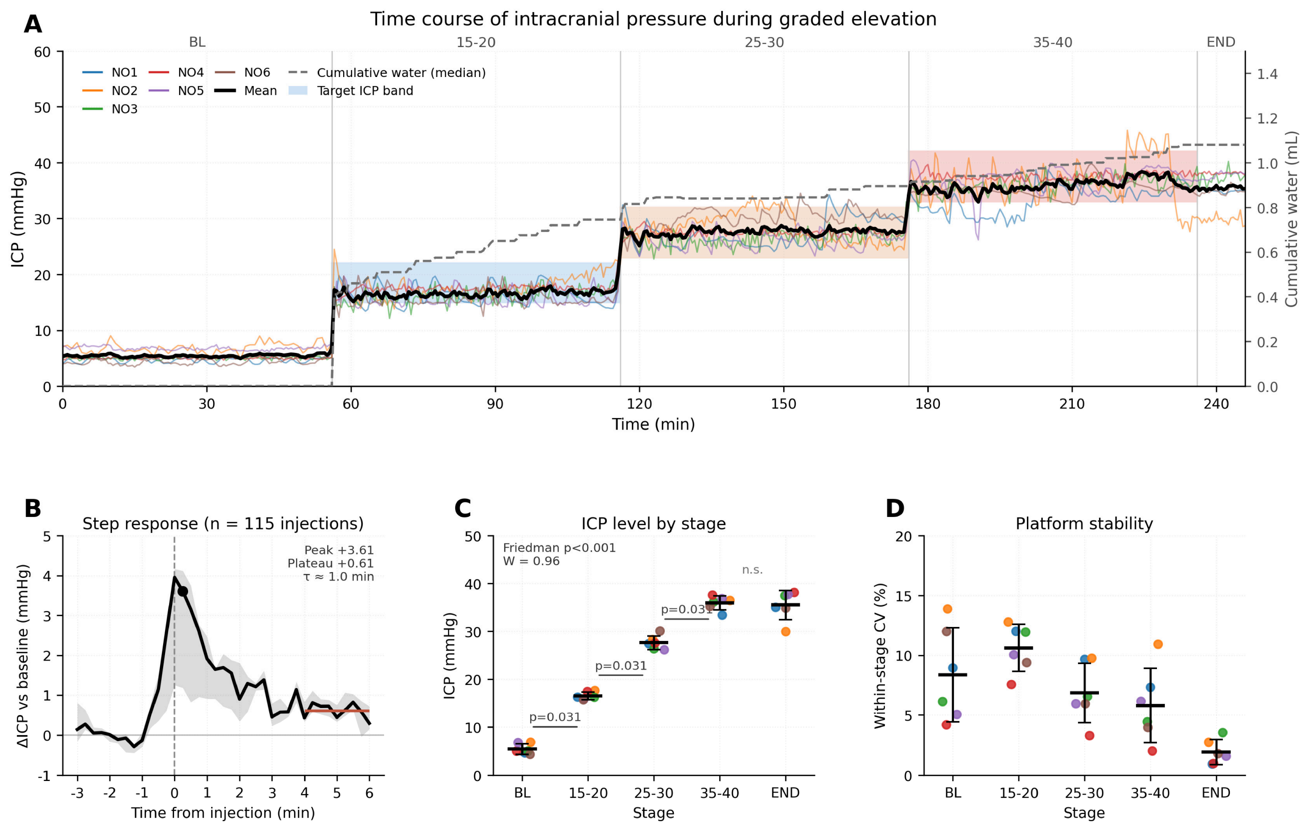

A
Time course of intracranial pressure during graded elevation
BL
15-20
25-30
35-40
END
NO1
NO2
NO3
NO4
NO5
NO6
Mean
Cumulative water (median)
Target ICP band
ICP (mmHg)
Cumulative water (mL)
Time (min)
B
Step response (n = 115 injections)
Peak +3.61
Plateau +0.61
τ ≈ 1.0 min
ΔICP vs baseline (mmHg)
Time from injection (min)
C
ICP level by stage
Friedman p<0.001
W = 0.96
p=0.031
p=0.031
p=0.031
n.s.
ICP (mmHg)
Stage
D
Platform stability
Within-stage CV (%)
Stage
