# Supplementary material for: Dynamic Evolution of Quantitative Electroencephalography During Graded Intracranial Hypertension and Screening of Candidate Early-Warning Features in a Rabbit Model
Source: Brain Sci. 2026 Oct 16;16(10):1116. doi: 10.3390/brainsci16101116 (PMC13702719; doi:10.3390/brainsci16101116)
Supplement: Supplementary file 1 [file brainsci-16-01116-s001.zip › brainsci-4598378-tex.zip!/Definitions/brainsci-4598378-g006.pdf]

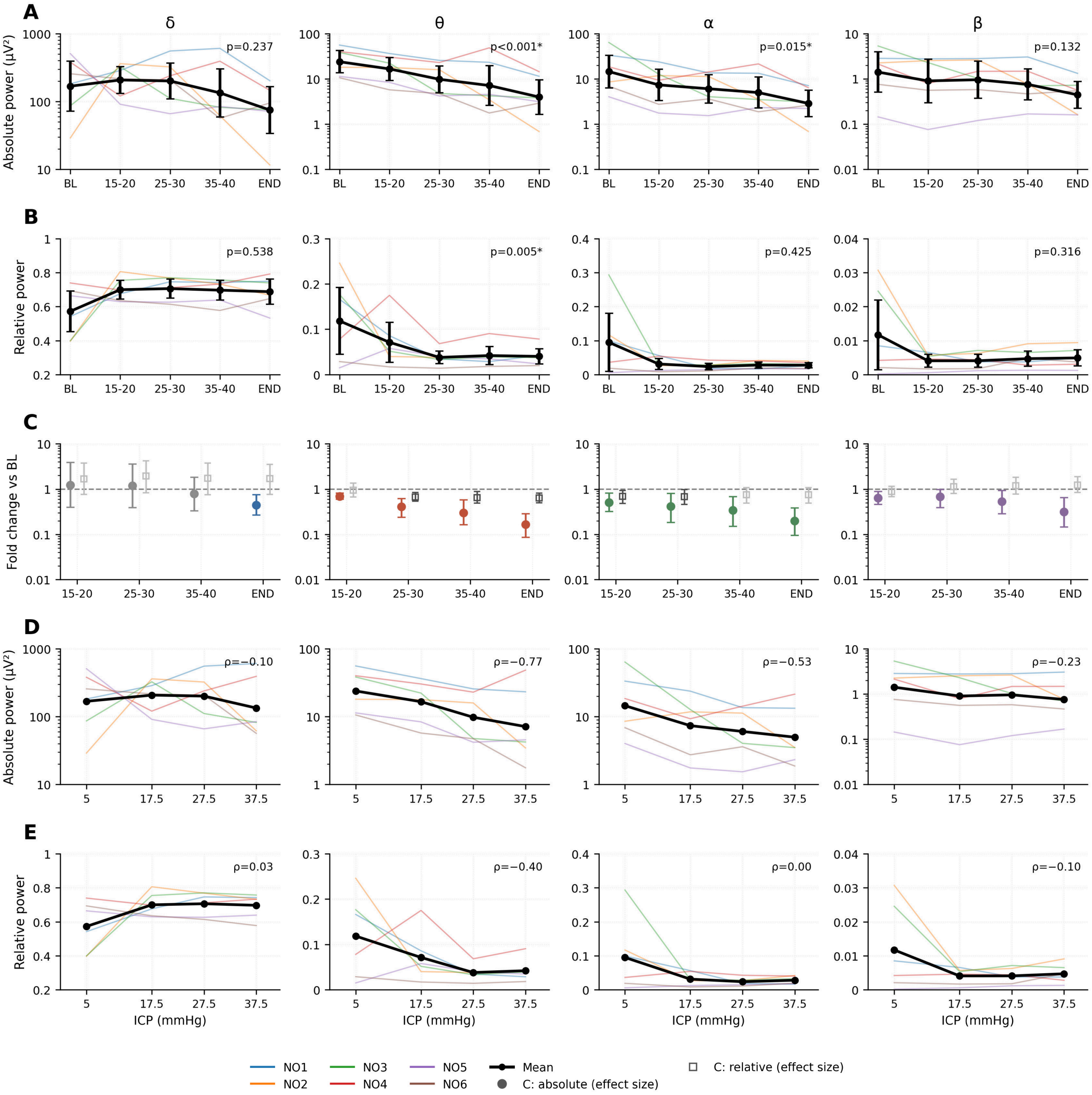

A
B
C
D
E
δ
θ
α
β
Absolute power (μV²)
Relative power
Fold change vs BL
ICP (mmHg)
BL
15-20
25-30
35-40
END
p=0.237
p<0.001*
p=0.015*
p=0.132
p=0.538
p=0.005*
p=0.425
p=0.316
ρ=−0.10
ρ=−0.77
ρ=−0.53
ρ=−0.23
ρ=0.03
ρ=−0.40
ρ=0.00
ρ=−0.10
NO1
NO2
NO3
NO4
NO5
NO6
Mean
C: absolute (effect size)
C: relative (effect size)
