# Supplementary material for: Dynamic Evolution of Quantitative Electroencephalography During Graded Intracranial Hypertension and Screening of Candidate Early-Warning Features in a Rabbit Model
Source: Brain Sci. 2026 Oct 16;16(10):1116. doi: 10.3390/brainsci16101116 (PMC13702719; doi:10.3390/brainsci16101116)
Supplement: Supplementary file 1 [file brainsci-16-01116-s001.zip › brainsci-4598378-tex.zip!/Definitions/brainsci-4598378-g007.pdf]

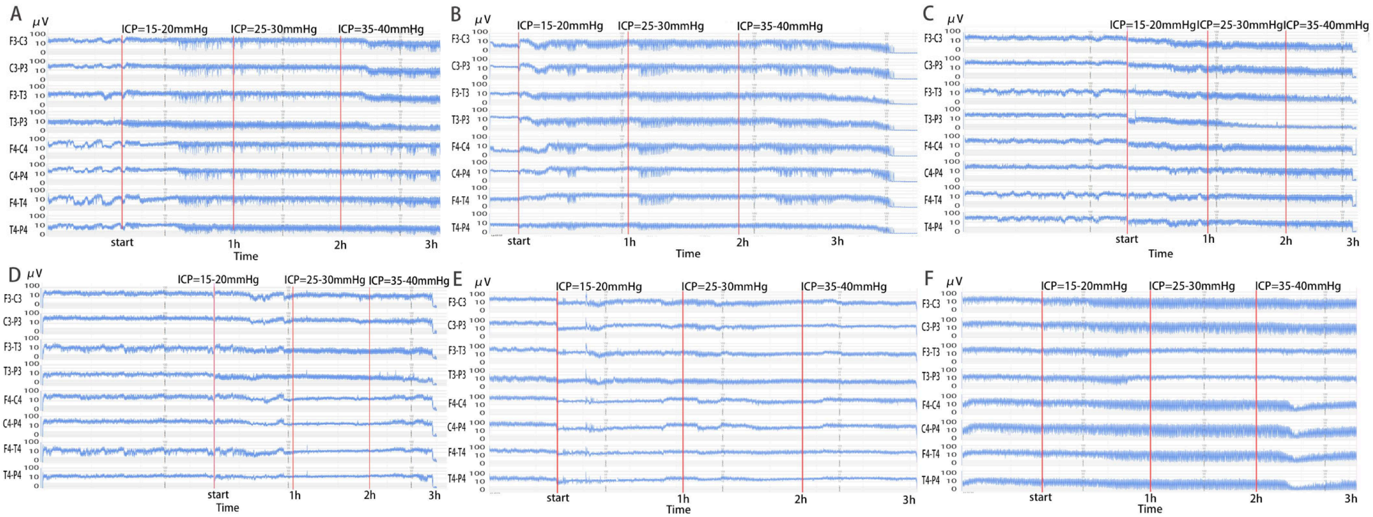

A
B
C
D
E
F
μV
ICP=15-20mmHg
ICP=25-30mmHg
ICP=35-40mmHg
F3-C3
C3-P3
F3-T3
T3-P3
F4-C4
C4-P4
F4-T4
T4-P4
100
10
0
start
1h
2h
3h
Time
