# Supplementary material for: Dynamic Evolution of Quantitative Electroencephalography During Graded Intracranial Hypertension and Screening of Candidate Early-Warning Features in a Rabbit Model
Source: Brain Sci. 2026 Oct 16;16(10):1116. doi: 10.3390/brainsci16101116 (PMC13702719; doi:10.3390/brainsci16101116)
Supplement: Supplementary file 1 [file brainsci-16-01116-s001.zip › brainsci-4598378-tex.zip!/Definitions/brainsci-4598378-g008.pdf]

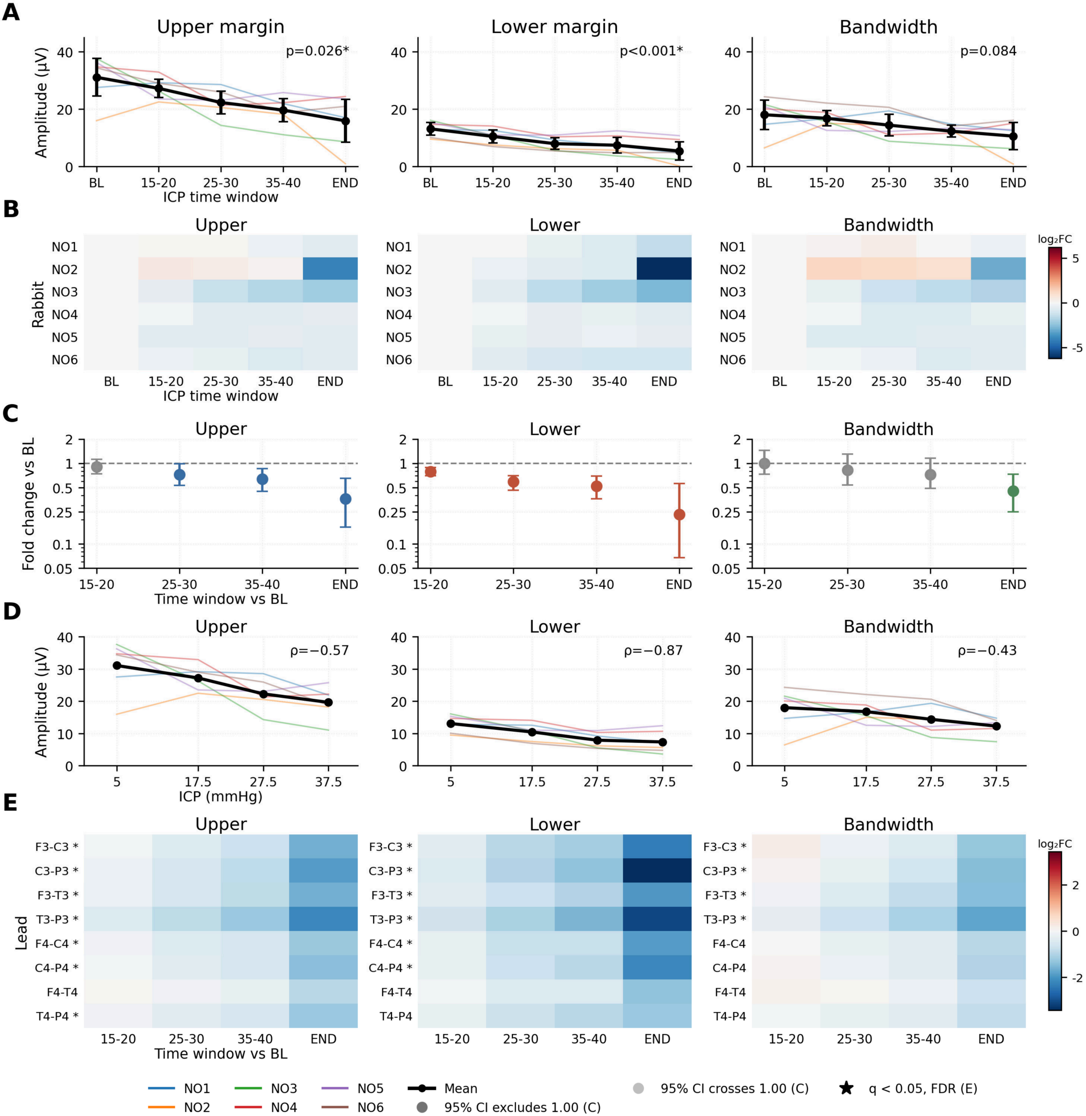
A
Upper margin
Lower margin
Bandwidth
p=0.026*
p<0.001*
p=0.084
Amplitude (μV)
ICP time window
BL
15-20
25-30
35-40
END
B
Upper
Lower
Bandwidth
Rabbit
NO1
NO2
NO3
NO4
NO5
NO6
log₂FC
C
Fold change vs BL
Time window vs BL
D
ρ=−0.57
ρ=−0.87
ρ=−0.43
ICP (mmHg)
E
Lead
F3-C3 *
C3-P3 *
F3-T3 *
T3-P3 *
F4-C4 *
C4-P4 *
F4-T4
T4-P4 *
F4-C4
C4-P4
T4-P4
Mean
95% CI crosses 1.00 (C)
95% CI excludes 1.00 (C)
q < 0.05, FDR (E)
