# Supplementary material for: Dynamic Evolution of Quantitative Electroencephalography During Graded Intracranial Hypertension and Screening of Candidate Early-Warning Features in a Rabbit Model
Source: Brain Sci. 2026 Oct 16;16(10):1116. doi: 10.3390/brainsci16101116 (PMC13702719; doi:10.3390/brainsci16101116)
Supplement: Supplementary file 1 [file brainsci-16-01116-s001.zip › brainsci-4598378-tex.zip!/Definitions/brainsci-4598378-g009.pdf]

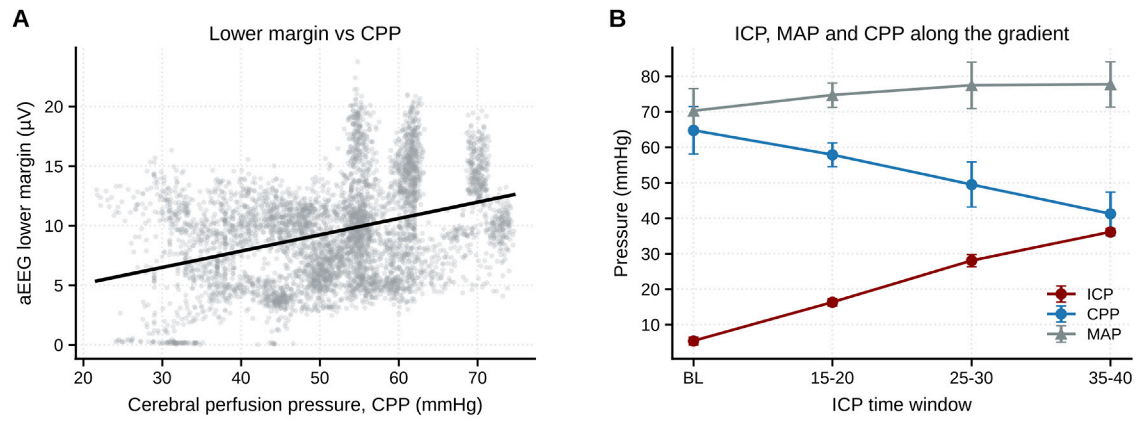

A
Lower margin vs CPP
aEEG lower margin (µV)
0
5
10
15
20
20
30
40
50
60
70
Cerebral perfusion pressure, CPP (mmHg)
B
ICP, MAP and CPP along the gradient
Pressure (mmHg)
10
20
30
40
50
60
70
80
BL
15-20
25-30
35-40
ICP time window
ICP
CPP
MAP
