# Supplementary material for: Dynamic Evolution of Quantitative Electroencephalography During Graded Intracranial Hypertension and Screening of Candidate Early-Warning Features in a Rabbit Model
Source: Brain Sci. 2026 Oct 16;16(10):1116. doi: 10.3390/brainsci16101116 (PMC13702719; doi:10.3390/brainsci16101116)
Supplement: Supplementary file 1 [file brainsci-16-01116-s001.zip › brainsci-4598378-tex.zip!/Definitions/brainsci-4598378-g010.pdf]

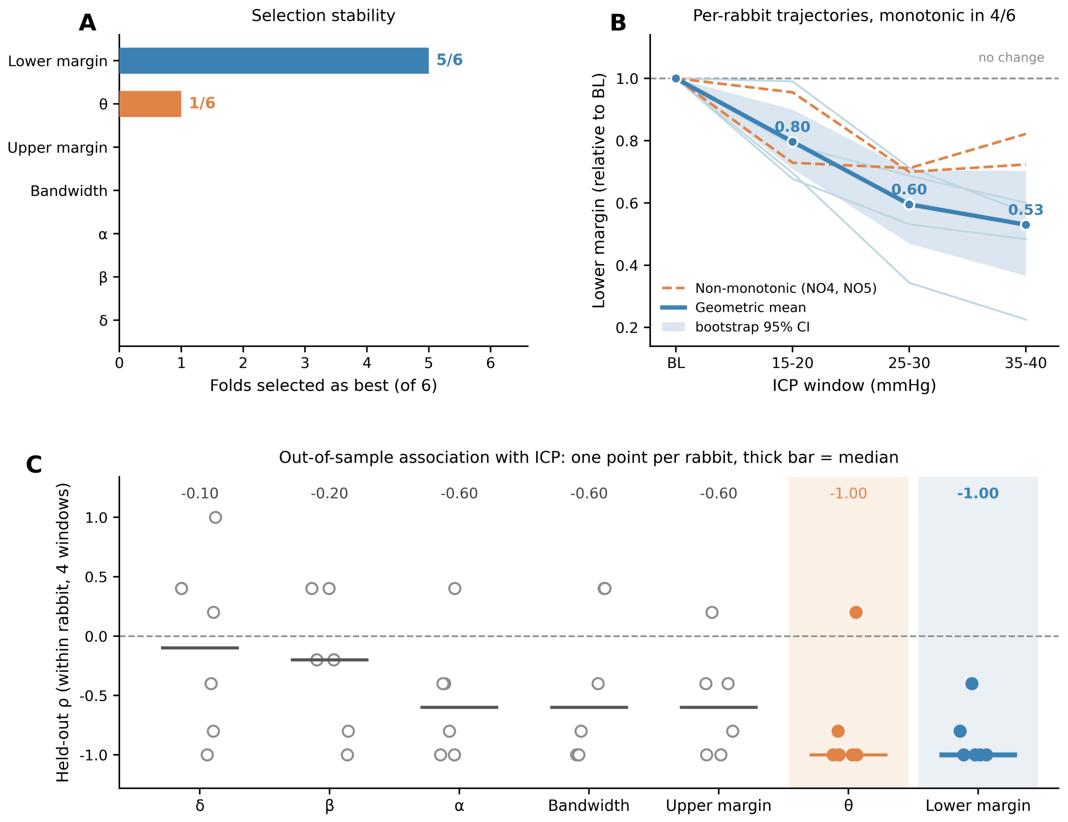

A
Selection stability
Lower margin
θ
Upper margin
Bandwidth
α
β
δ
5/6
1/6
0
1
2
3
4
5
6
Folds selected as best (of 6)
B
Per-rabbit trajectories, monotonic in 4/6
Lower margin (relative to BL)
no change
1.0
0.8
0.6
0.4
0.2
0.80
0.60
0.53
Non-monotonic (NO4, NO5)
Geometric mean
bootstrap 95% CI
BL
15-20
25-30
35-40
ICP window (mmHg)
C
Out-of-sample association with ICP: one point per rabbit, thick bar = median
Held-out ρ (within rabbit, 4 windows)
-0.10
-0.20
-0.60
-0.60
-0.60
-1.00
-1.00
1.0
0.5
0.0
-0.5
-1.0
δ
β
α
Bandwidth
Upper margin
θ
Lower margin
