# Supplementary material for: Dynamic Evolution of Quantitative Electroencephalography During Graded Intracranial Hypertension and Screening of Candidate Early-Warning Features in a Rabbit Model
Source: Brain Sci. 2026 Oct 16;16(10):1116. doi: 10.3390/brainsci16101116 (PMC13702719; doi:10.3390/brainsci16101116)
Supplement: Supplementary file 1 [file brainsci-16-01116-s001.zip › brainsci-4598378-tex.zip!/Definitions/logo-updates-eps-converted-to.pdf]

Check for updates
